# Watercraft decontamination practices to reduce the viability of aquatic invasive species implicated in overland transport

**DOI:** 10.1038/s41598-023-33204-0

**Published:** 2023-05-04

**Authors:** Shrisha Mohit, Timothy B. Johnson, Shelley E. Arnott

**Affiliations:** 1 Department of Biology, Queen’s University, 116 Barrie Street, Kingston, ON K7L 3N6 Canada; 2 Ontario Ministry of Northern Development, Mines, Natural Resources, and Forestry, Glenora Fisheries Station, Picton, ON K0K 2T0 Canada

**Keywords:** Ecology, Ecology

## Abstract

Recreational boating activities enable aquatic invasive species (AIS) dispersal among disconnected lakes, as invertebrates and plants caught on or contained within watercraft and equipment used in invaded waterbodies can survive overland transport. Besides simple preventive measures such as "clean, drain, dry", resource management agencies recommend decontaminating watercraft and equipment using high water pressure, rinsing with hot water, or air-drying to inhibit this mode of secondary spread. There is a lack of studies assessing the efficacy of these methods under realistic conditions and their feasibility for recreational boaters. Hence, we addressed this knowledge gap via experiments on six invertebrate and plant AIS present in Ontario. Washing at high pressures of 900–1200 psi removed the most biological material (90%) from surfaces. Brief (< 10 s) exposure to water at ≥ 60 °C caused nearly 100% mortality among all species tested, except banded mystery snails. Acclimation to temperatures from 15 to 30 °C before hot water exposure had little effect on the minimum temperature required for no survival. Air-drying durations producing complete mortality were ≥ 60 h for zebra mussels and spiny waterfleas, and ≥ 6 days among plants, whereas survival remained high among snails after a week of air-drying. Hot water exposure followed by air-drying was more effective than either method separately against all species tested.

## Introduction

Invasive species have been introduced to aquatic ecosystems through various pathways, such as the discharge of ballast waters, the release or escape of species intended for the aquarium or water garden trade and live food market, the use and transport of live baits, as well as the movement of watercraft among disconnected waterbodies^1–3^. There is ample evidence that recreational boating activities are associated with the secondary spread of aquatic invasive species (AIS) among lakes, as the transport of boats and other equipment enable these species to overcome land barriers^4–6^. Recreational boats and gear used in infested waters are apt to catch or trap plant and invertebrate AIS on various structures such as boat trailers, ropes, anchors, anchor lines and nets, or in compartments that may retain water such as bilge and live wells^5,7–9^. Unless they are removed, these AIS may survive overland transport^10,11^, whether in humid areas such as the bilge, live wells, and bait buckets, among macrophytes or sediments attached to boats and trailers^5,8,12,13^, or owing to physiologic or metabolic abilities to tolerate conditions outside of water, until they are reintroduced into an aquatic environment^12,14–16^. Hence, recreational watercraft and equipment can become the vector for secondary spread of AIS to disconnected lakes and other water bodies^3^. With recreational boaters and lake users moving greater distances and more frequently among water bodies during peak seasons, there are potentially more occasions where AIS propagules are introduced to new environments in this manner^8,17–20^. It is therefore crucial to reduce the number of live or viable AIS propagules arriving at non-colonised sites to prevent their establishment and range expansion^21–23^.

To prevent the spread of AIS, numerous resource management agencies recommend that recreational boaters adopt practices to clean, drain, and dry watercraft and equipment before transport and use at another site^24,25^. For instance, in Ontario, the methods prescribed by the Ministry of Northern Development, Mines, Natural Resources and Forestry include washing with water at a pressure of 250 psi, rinsing with hot water at greater than 50 °C, or allowing all parts to air-dry for two to seven days^26^. The effect of similar and commonly recommended decontamination methods for recreational watercraft and equipment on different AIS have been previously studied, but there is no clear consensus for a broad-spectrum method that could be implemented by recreational boaters^24^. While air-drying was extensively studied, experiments on hot water use varied largely with regards to the application method and species assessed, whereas few studies evaluated pressure-washing and the use of cleaning products.

Air-drying is an inexpensive and low effort means of decontamination, and in general, increasing its duration lowers survival among various species. Some AIS exhibit high mortality after air-drying for up to 7 days, but others survived air-drying periods lasting weeks to months^24,27,28^. However, over 90% of all air-drying studies were conducted under laboratory conditions, thereby not accurately simulating the environmental variability that boaters would encounter when air-drying their boats and equipment over the span of several days^24^. Past studies have also found that hot water immersion at 50 °C for > 15 min resulted in total mortality among many species^27,29–31^. Similar results could be achieved with shorter exposure duration but only if higher water temperatures were used, such as hot water sprays at 60 °C, or steam sprays (≥ 100 °C)^30,32–34^. Despite evidence that hot water could be an effective decontamination tool, its application would be impractical under field conditions for recreational boaters and for large-scale decontamination such as the surface of boat hulls; not only would it require specific equipment capable of delivering and sustaining high water temperatures easily affected by environmental conditions^34,35^, but it also presents elevated risks of injury to the user and damage to the surrounding environment. No study to date has assessed the effect of hot water on aquatic invasive snails, despite the latter’s known tolerance to other decontamination methods^24^. Pressure-washing was among the less well-studied methods. Previous experiments assessing the efficacy of pressurised hot water^34,35^ did not include comparison groups testing different pressures, but rather, they primarily evaluated different water temperatures. However, studies by Rothlisberger et al.^20^ and Wong et al.^38^ evaluated the efficacy of pressure itself, comparing high and low water pressure groups. Rothlisberger et al. reported that 1800 psi removed significantly more entangled plants and small organisms than 40 psi, while Wong et al. demonstrated that 3000 psi removed dreissenid mussels from heavily encrusted surfaces faster than 1500 psi. Nevertheless, neither tested a range of pressures and, considering the variation in pressure output of pressure-washers found in retail, the efficacy of this method is thus not well understood. There is also a lack of research on the effects of implementing multiple means of decontamination sequentially or simultaneously. The extent to which combining different decontamination methods can improve efficacy, or if their effect differs across various species under the same conditions, is unknown. In addition, no study so far seems to have assessed whether the response of AIS to currently recommended decontamination methods is affected by acclimation to the changing environmental conditions over the span of a boating season. Previous research has shown that cold-acclimated invasive apple snails were more resistant to desiccation^36^, and invasive marine macrophytes were more resilient than native species after exposure to heat stress if they had been previously acclimated to warmer conditions^37^.

Informed by the gaps in knowledge identified in the literature and considering environmental and logistic factors that recreational boaters may encounter when decontaminating boats and equipment, we performed a series of experiments to test the efficacy of pressure-washing, brief hot water exposure, air-drying in outdoor conditions, and the combination of hot water exposure followed by air-drying. The goal of our study was to reveal empirical information that could support or inform modifications to recommended decontamination measures. Our pressure washing experiments on periphyton and plant fragments tested pressures ranging from the equivalent of no washing to those that could be generated using retail electric washers, on surfaces simulating a metal boat hull. Similarly, the experiments on the efficacy of hot water application and air-drying consisted of trials simulating brief hot-water exposure akin to rinsing rather than soaking, and air-drying periods representing realistic durations between watercraft and equipment use at different sites, respectively. Additionally, we designed experiments with hot water exposure followed by air-drying to determine if combining decontamination methods could have an additive or synergistic effect on efficacy. To identify the most effective conditions against diverse species, the experiments were conducted on three invertebrate and three plant AIS present in Ontario, namely zebra mussels (*Dreissena polymorpha*), banded mystery snails (*Viviparus georgianus*), spiny waterfleas (*Bythotrephes cederstroemi*), Eurasian watermilfoil (*Myriophyllum spicatum*), Carolina fanwort (*Cabomba caroliniana*), and European frogbit (*Hydrocharis morsus-ranae*). We also assessed whether acclimation to different water temperatures affected the viability of AIS subjected to the hot water treatment.

## Results

### Results of pressure-washing experiments

**Figure 1:**
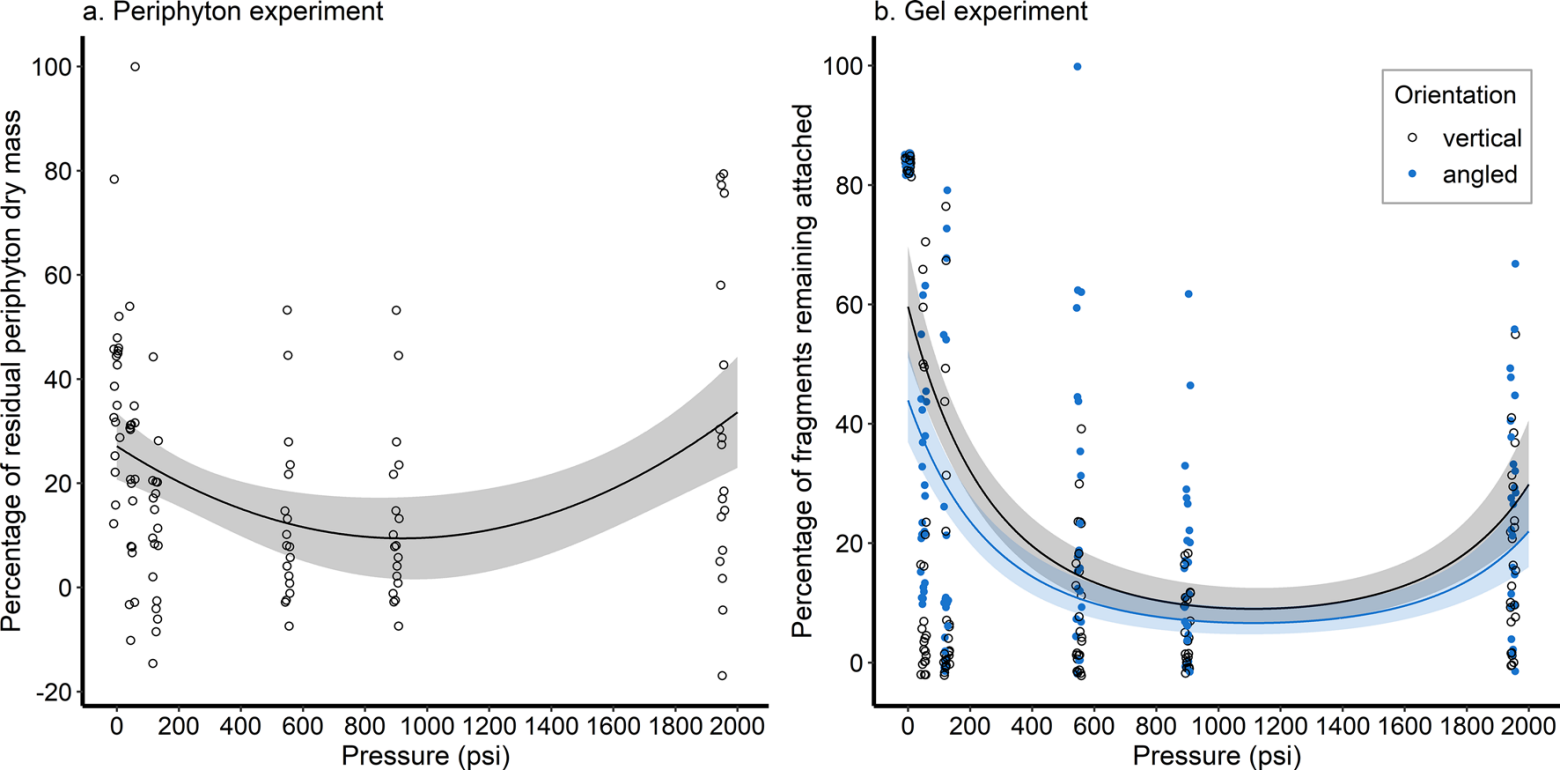
Relationship between amount of material remaining on surfaces and water pressure for (**a**) naturally colonized and (**b**) gel seeded experiments. Jittered points represent the observed data, solid lines and shaded areas indicate regression lines and 95% confidence bands, respectively. Open black circles correspond to tiles placed vertically or perpendicular to the ground, and blue dots to tiles placed at a 20° angle to the ground.

Increasing pressure significantly reduced the dry mass of matter (periphyton) remaining on washed surfaces except at the highest pressure tested (1950 psi), where the amount of residue increased compared to lower pressures, regardless of surface orientation (Fig. 1a; Supplementary Table S1). Using the best fit regression model, we predicted that a pressure of 921 psi removed the most periphyton from the tiles (90.6%). We obtained similar results from the experiment replacing periphyton with water-based gel; while there was no interaction between orientation and pressure based on the best fitted model, the number of leaves and/or fragments of leaves remaining attached to the gel significantly decreased as pressure increased, except at 1950 psi, in both orientation groups (Fig. 1b). Orientation also had a significant effect on the number of leaves remaining, with a vertical surface retaining 36% more than a surface angled at 20° (Supplementary Table S1). We estimated that a pressure of 1113 psi removed the greatest number of leaves and fragments by pressure-washing, for either the vertical (89.2%) or angled surface (92.0%).

### Results of experiments on invertebrates

#### Effects of hot water immersion on invertebrates

**Figure 2:**
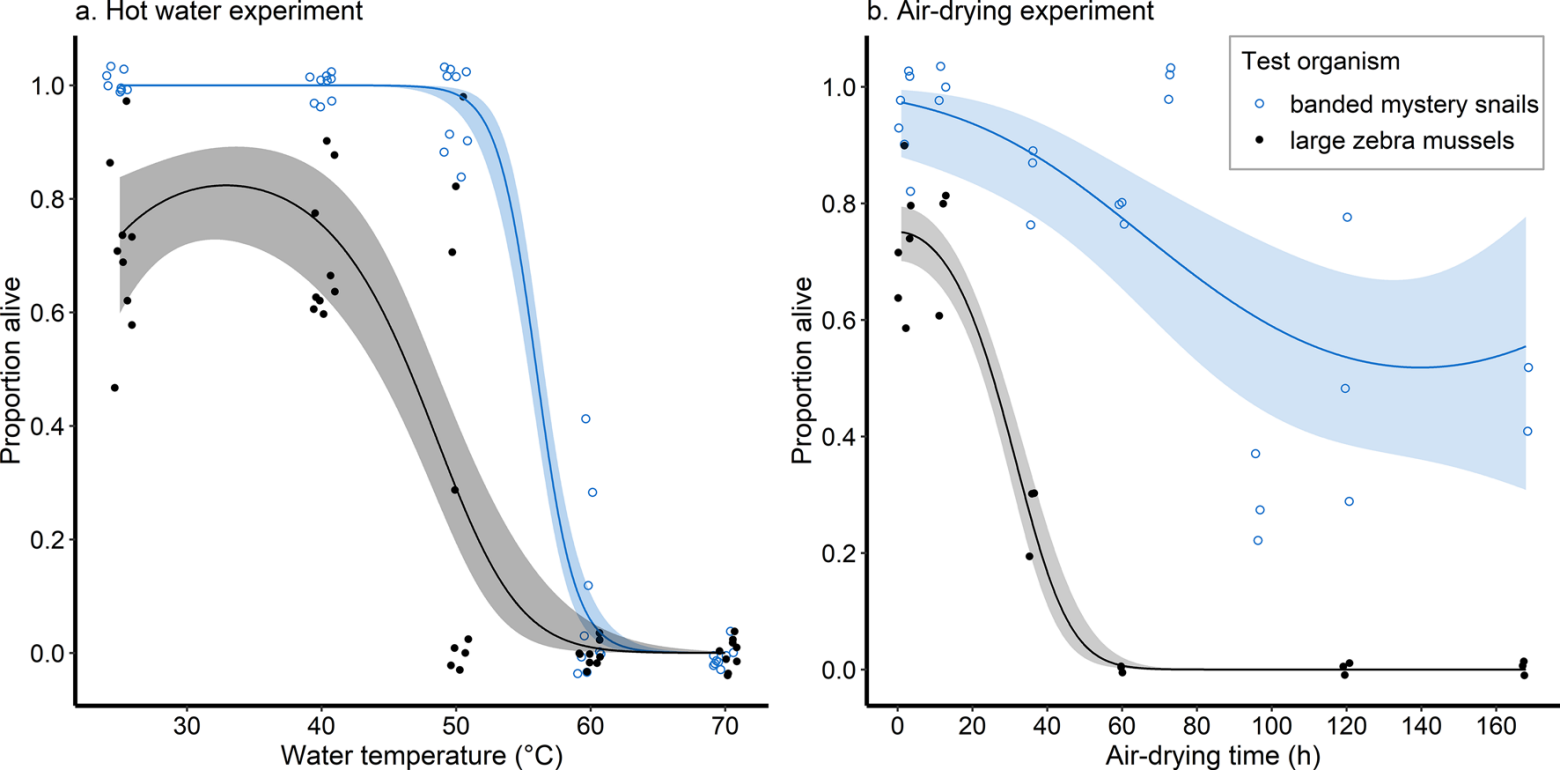
Relationship between banded mystery snail and large zebra mussel survival and (**a**) hot water temperature (only results for 5 s immersion are shown) and (**b**) air-drying duration. Open blue circles and black dots correspond to banded mystery snails and large zebra mussels, respectively.

**Figure 3:**
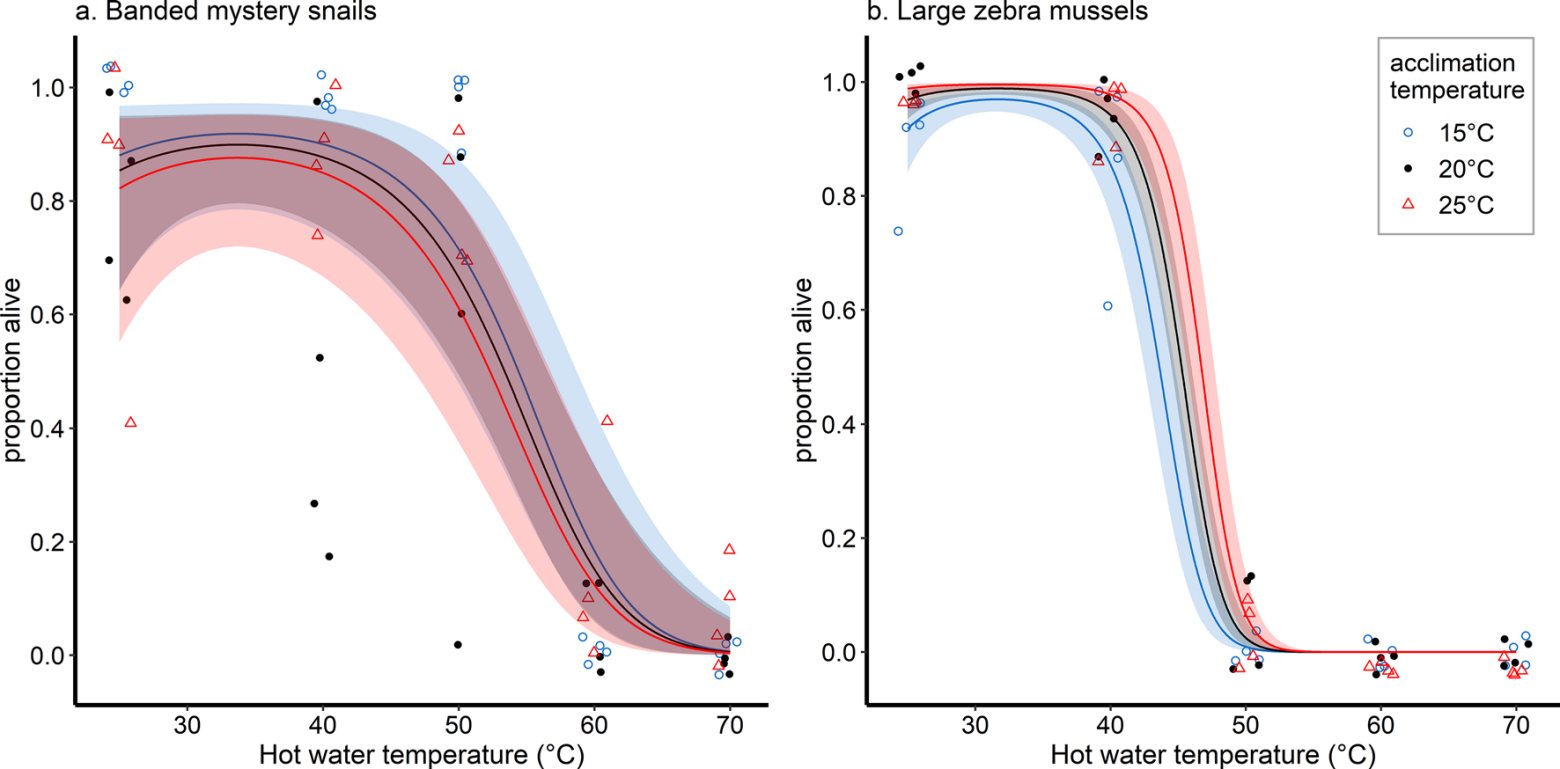
Relationship between (**a**) banded mystery snail and (**b**) zebra mussel survival and hot water temperature at three acclimation temperatures. Open blue circles, black dots, red triangles correspond to acclimation at 15 °C, 20 °C, and 25 °C, respectively.

When exposed to hot water, the number of banded mystery snails that survived decreased significantly with either increasing water temperature or increasing exposure duration (Fig. 2a, Supplementary Table S2). We also observed a rapid decrease in survival at temperatures ≥ 50 °C and estimated that a minimum water temperature of 65 °C, 63 °C, and 59 °C was required for 99% mortality when snails are exposed to hot water for 2 s, 5 s, and 10 s, respectively. From the acclimation experiments where only an exposure of 5 s was applied, we found that increasing acclimation temperature produced no statistically significant increase or decrease in snail survival, whereas increasing the water temperature for immersion significantly reduced snail survival (Fig. 3a).

Among zebra mussels of both size classes, increasing water temperature and immersion duration reduced survival, with a rapid decrease following immersion at 40 °C (Fig. 2a). Increasing water temperature resulted in significantly fewer small zebra mussels surviving after treatment whereas the effect of immersion duration was not statistically significant (Supplementary Table S3), and we predicted that a temperature of approximately 57 °C could produce 99% mortality. However, among large zebra mussels, increasing both water temperature and immersion time significantly reduced survival, with higher temperatures of 58 °C (10 s), 60 °C (5 s), and 61 °C (2 s) needed to produce 99% mortality. Our acclimation experiments on large zebra mussels revealed that increasing acclimation temperature led to significantly higher survival after treatment, as opposed to increasing water temperature which significantly reduced survival, again with a rapid decrease at temperatures above 40 °C (Fig. 3b). Nonetheless, the difference in hot water temperatures producing 99% mortality differed by 2 °C at most among the three acclimation groups (50 °C, 51 °C, and 52 °C for the 15 °C, 20 °C, and 25° acclimation groups, respectively), albeit being lower than in the experiment without acclimation.

**Figure 4:**
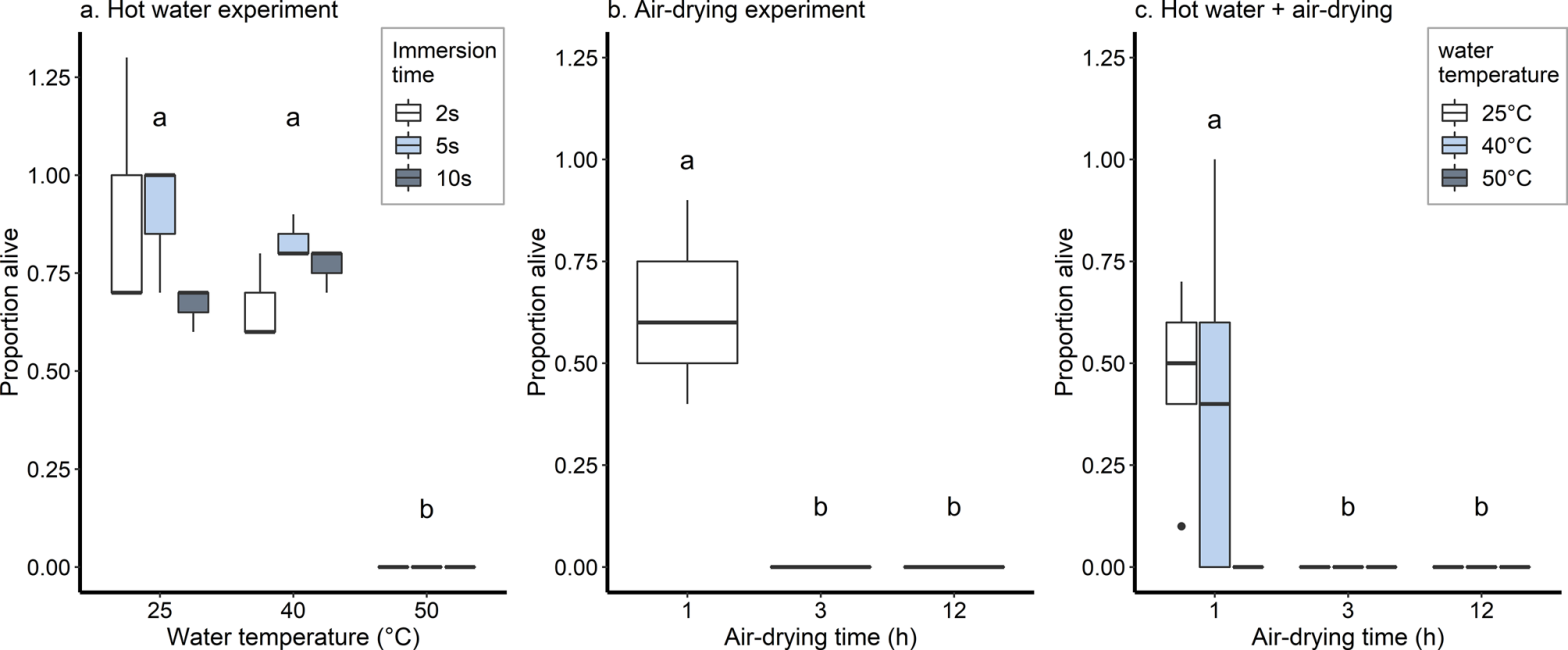
Spiny waterflea survival at different (**a**) immersion temperatures, (**b**) air-drying durations, and (**c**) combined temperature plus air-drying. Groups sharing the same letter indicators were not significantly different from one another based on pairwise Tukey HSD test. White, light blue, and dark grey boxes correspond to immersion durations of 2 s, 5 s, and 10 s, respectively, in panel *a*, and to water temperatures of 25 °C, 40 °C, and 50 °C in panel *c*.

Spiny waterfleas exposed to hot water did not survive temperatures ≥ 50 °C and we found only a significant main effect of water temperature on survival (two-way ANOVA, F = 91.241, df_temperature_ = 2, df_residuals_ = 22, *p* < 0.05). Significantly fewer spiny waterfleas survived after immersion at 50 °C than at either 25 °C or 40 °C (Tukey HSD adjusted *p* < 0.05), whereas there was no statistically significant difference in the number of surviving spiny waterfleas at 25 °C compared to 40 °C (Tukey HSD adjusted *p* = 0.593; Fig. 4a).

#### Effects of air-drying on invertebrates

The number of banded mystery snails surviving decreased significantly as air-drying duration increased (Fig. 2b; Supplementary Table S4). The rate of decrease in survival was progressively diminished as time increased; however, the maximum mortality achieved among banded mystery snails was 48.2%, which would result from a minimum air-drying duration of 140 h as per the best-fitted regression model for our data, where the maximum air-drying duration tested was 7 days. Zebra mussels were more susceptible to air-drying than snails, with some observed differences between the two size classes. Among small zebra mussels, survival was high and variable up to 12 h of air-drying, but then dropped rapidly such that no small zebra mussels were alive in the 36 h group onwards (Supplementary Figure S1). Our best model for the data revealed no statistically significant decrease in survival with increasing air-drying duration, but these results should be interpreted with caution due to the lack of variation in the dataset for air-drying durations ≥ 36 h. Survival among the large zebra mussel size class significantly decreased with prolonged air-drying (Fig. 2b), with a minimum of 58 h required for 99% mortality.

The results of the one-way ANOVA for spiny waterfleas showed that air-drying duration had a significant effect on the number of spiny waterfleas remaining alive after exposure to air (F = 19.00, df_time_ = 2, df_residuals_ = 6, *p* = 0.003). Significantly fewer spiny waterfleas were alive after 3 h (Tukey HSD adjusted *p* = 0.004) and 12 h (Tukey HSD adjusted *p* = 0.004) of air-drying compared to after 1 h. There was 100% mortality among spiny waterfleas after > 3 h of air-drying (Fig. 4b).

#### Effects of combining hot water exposure and air-drying on invertebrates

When used in combination, increasing both water temperature and air-drying duration resulted in significantly higher snail mortality, with water temperature having a greater effect than drying time (Supplementary Table S5). As longer air-drying duration resulted in reduced survival for all temperature groups, we predicted that after hot water immersion at < 60 °C, air-drying for 72 h (25 °C), 97 h (40 °C) and 66 h (50 °C) was needed to produce 50% mortality (Table 1).

**Table 1:** Predicted air-drying time required for 50–99% banded mystery snail mortality after hot water immersion for 5 s at temperatures from 25 to 70 °C.

Mortality	Water temperature				
	25 °C	40 °C	50 °C	60 °C	70 °C
	Air-drying duration				
50%	72.10 h	96.93 h	66.13 h	†	†
	3.0 days	4.0 days	2.8 days		
90%	*	*	*	82.41 h	†
				3.4 days	
99%	*	*	*	*	68.47 h
					2.9 days

*Air-drying duration cannot be accurately predicted for these mortality rates and water temperatures from our best fit model.

^†^Mortality rates expected without air-drying (< 3 h) at these temperatures.

We conducted the analyses for both zebra mussel size classes only on data from the 25 and 40 °C groups as we recorded complete mortality prior to air-drying at water temperatures ≥ 50 °C in these experiments. Mortality among both large and small mussels was significantly higher by increasing air-drying duration at lower temperatures (Supplementary Table S5), with a rapid decrease in survival after air-drying for at least 12 h. We estimated that a minimum duration of 27.5 h and 40.2 h were required for 99% mortality among small and large zebra mussels, respectively, following exposure to water temperatures from 25 to < 50 °C.

The results of our two-way ANOVA also showed that spiny waterflea survival was reduced as air-drying duration increased following hot water immersion. We did not detect any significant interaction between water temperature and air-drying time on spiny waterflea survival (F = 1.626, df_interaction_ = 4, df_residuals_ = 22, *p* = 0.203). However, only the main effect of air-drying duration (F = 8.891, df_time_ = 2, df_residuals_ = 24, *p* = 0.001), but not water temperature (F = 1.725, df_temperature_ = 2, df_residuals_ = 24, *p* = 0.198), was statistically significant, and a greater number of spiny waterfleas survived in the group exposed to 1 h air-drying after hot water immersion, than in the 3 h and 12 h groups, where we observed complete mortality (Tukey HSD adjusted *p* = 0.004; Fig. 4c).

### Results of experiments on macrophytes

#### Effects of hot water on macrophytes

**Figure 5:**
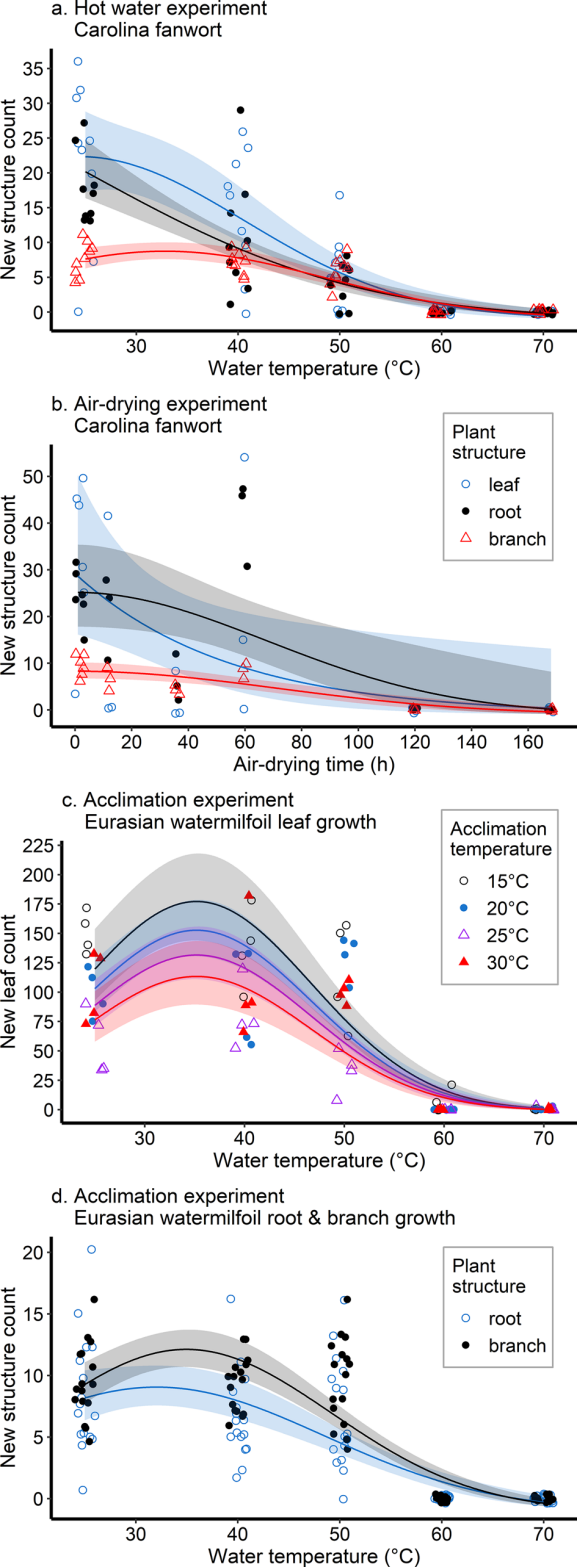
Relationship between new structure growth after 4 weeks (or 3 weeks for acclimation experiment) and (**a**) temperature, (**b**) air-drying, (**c**) and (**d**) temperature following acclimation for the most resistant macrophyte species within each experiment type. Panels *a* and *b* (results for Carolina fanwort): open blue circles—number of leaves, black dots—number of roots, and red triangles—number of branches. Panel *c* (acclimation experiment results for Eurasian watermilfoil): open black circle—acclimation at 15 °C, blue dot—at 20 °C, open purple triangle—at 25 °C, and filled red triangle—at 30 °C. Panel *d*: open blue circles—number of roots, and black dots—number of branches.

Our results revealed that among the three aquatic plant species tested, increasing water temperature significantly decreased new leaf, root, branch or turion growth (Supplementary Tables S6 to S8). Although the selected models indicate that root growth in Carolina fanwort and turion production in European frogbit was negatively associated with immersion time, this effect was not statistically significant. In general, there was a steady decline in the emergence of new structures with exposure to hot water at temperatures ≥ 40 °C for all species (Fig. 5a, Supplementary Figures S2 to S4). We determined that new growth can be prevented by brief exposure to hot water at 58 °C among Eurasian watermilfoil, 58 °C to 62 °C among Carolina fanwort, and 35 °C to 42 °C among European frogbit. While the acclimation experiments on Eurasian watermilfoil also showed that increasing hot water temperature significantly decreases growth, interestingly we found a significant inverse relationship between acclimation temperature and leaf growth (Fig. 5c), but not root and branch development (Fig. 5d). We predicted that minimum temperatures of approximately 63 °C would be required to prevent new root and branch growth irrespective of acclimation temperature, but higher temperatures close to 70 °C would prevent new leaf growth among fragments across all acclimation temperatures.

#### Effects of air-drying on macrophytes

Increasing air-drying duration significantly decreased production of new structures among all three macrophytes (Fig. 5b, Supplementary Table S9 and Supplementary Figures S5 to S6). However, tolerance to desiccation differed considerably among the three species tested, with Carolina fanwort requiring higher minimum air-drying durations to prevent new growth (leaf, 147 h; root, 153 h; branch, 126 h) than Eurasian watermilfoil (leaf, 84 h; root, 77 h; branch, 84 h), and with European frogbit being especially susceptible to desiccation (leaf, 1 h; turion, 8.9 h).

#### Effects of hot water immersion followed by air-drying on macrophytes

Combining decontamination methods generally produced a rapid decline in new growth among aquatic macrophytes (Supplementary Table S10 and Supplementary Figures S7 to S9). Among Eurasian watermilfoil and Carolina fanwort fragments, increasing both hot water temperature and air-drying duration resulted in significantly reduced new leaf, root, and branch growth. Although we also found a statistically significant positive interaction between hot water temperature and air-drying duration among Carolina fanwort, its magnitude was smaller than the significant negative main effects of water temperature and air-drying. Combining the two techniques drastically reduced the air-drying duration required for no growth in Eurasian watermilfoil and Carolina fanwort compared to implementing air-drying only (Table 2).

**Table 2:** Minimum air-drying duration required for no new growth of leaves, roots, and branches of Eurasian watermilfoil and Carolina fanwort without and after hot water exposure.

Eurasian watermilfoil	Air-drying only	Hot water temperature				
		25 °C	40 °C	50 °C	60 °C	70 °C
	Duration	Air-drying duration				
Leaf	83.58 h	91.77 h	70.23 h	50.32 h	26.07 h	*
	3.5 days	3.8 days	2.9 days	2.1 days	1.1 days	
Root	76.89 h	48.56 h	32.63 h	24.20 h	16.82 h	10.14 h
	3.2 days	2.0 days	1.4 days	1.0 day	0.7 day	0.4 day
Branch	84.08 h	41.30 h	28.88 h	21.97 h	15.77 h	10.03 h
	3.5 days	1.7 days	1.2 days	0.9 day	0.7 day	0.4 day

Carolina fanwort	Duration	Air-drying duration				
Leaf	147.24 h	65.19 h	54.65 h	38.37 h	*	*
	6.1 days	2.7 days	2.3 days	1.6 days		
Root	153.12 h	60.86 h	53.01 h	38.25 h	*	*
	6.4 days	2.5 days	2.2 days	1.6 days		
Branch	125.54 h	59.22 h	50.67 h	35.44 h	*	*
	5.2 days	2.5 days	2.1 days	1.5 days		

*No new growth expected even without air-drying (or < 3 h) at these temperatures.

While neither water temperature nor air-drying duration had a significant effect on new European frogbit leaf growth, our models revealed that new turion growth increased significantly with increasing water temperature but was not affected by air-drying duration. However, the count of new structures was low across all replicates, and we could not accurately predict the conditions required to prevent new growth among European frogbit.

## Discussion

The findings from this study provide support for the use of hot water, air-drying, and pressure washing as decontamination methods for recreational watercraft, as recommended in Ontario and elsewhere. Our experiments were designed to bridge the gap in knowledge identified in the literature^24^ and assess the efficacy of decontamination methods with the potential to be safely carried out by recreational boaters. Our results revealed that specific conditions are nonetheless required to ensure the effectiveness of these decontamination methods in reducing the viability of aquatic invasive invertebrates or plants that could potentially be transported among lakes.

Two studies that assessed the efficacy of pressure-washing exclusively both demonstrated the efficacy of high pressure (≥ 1500 psi) in removing attached material from surfaces^20,38^. In their experiment evaluating the time taken for high pressures of 1500 psi and 3000 psi to remove encrusted mussels from boat hulls, Wong et al.^38^ employed pressures which may not be feasibly reproduced by recreational boaters. Rothlisberger et al.^20^ however, also included a low-pressure group (40 psi) and found that high water pressure (1800 psi) removed more attached plant material (83%) and small organisms (91%) than 40 psi (macrophytes, 62%; small organisms, 74%). By assessing a range of pressures, we determined that moderate water pressures from approximately 900 to 1200 psi, corresponding to the output of light-duty or electrical pressure-washers, were sufficient to eliminate about 90% of the amount of material attached to surfaces, whereas higher pressures did not have better efficacy. This could be explained by the highest pressure in our experiment causing more splash back and redistributing the dislodged material over the surface instead of the water running off as with the lower pressure groups. Nonetheless our study shows that commercially available medium-duty pressure-washers can be effective cleaning tools for recreational boaters. Our experiments however do present a few limitations that can be addressed in future studies. Firstly, while our setup using 20 × 20 cm metal tiles was intended to replicate the shape of a section of a boat hull, conducting the experiment on a model hull instead would be ideal, as it would provide a larger, more representative area for pressure washing. Moreover, the metal tiles used do not account for the properties of various materials and coatings used for boat hulls, which may potentially impact the efficacy of pressure washing. Finally, while we quantified pressure in PSI (pounds per square inch) as in previous studies and recommended decontamination guidelines, future studies could also include “cleaning units”, which is the product of the pressure washer flow rate (in gallons per minute, GPM) and PSI, thus standardising the variable representing pressure regardless of pressure washer specifications and water supply flow. Manipulating these two parameters could also enable the inclusion of a greater number of intermediate treatment groups.

When using hot water for decontamination—more specifically, brief exposure durations simulating rinsing rather than prolonged immersion—a minimum temperature of 60 °C was necessary to produce 99% mortality among small invertebrates and the plant species tested. In contrast, banded mystery snails were more resistant and required higher temperatures of ≥ 65 °C for complete mortality. We also assessed whether mortality rates would be influenced by seasonal changes in the lake water temperature experienced by the species of interest over the course of a boating season; we found that Eurasian watermilfoil and snails acclimated to cooler waters were more resistant to hot water exposure, in line with previous studies that found cold-acclimated snails to be more tolerant of various stressors^36,39,40^, whereas the opposite was noted among zebra mussels. Nonetheless, the minimum hot water temperature required for complete mortality only differed by approximately 2 °C among all species tested, suggesting that seasonal adjustments to the minimum water temperature would add unnecessary complexity to decontamination protocols, regardless of lake water temperature.

Overall, the findings of our study revealed that rinsing at water temperatures higher than the commonly recommended 50 °C is necessary for complete mortality among several aquatic invasive species, or alternatively, at lower temperatures for longer periods. However, watercraft decontamination outdoors using water at very high temperatures would be impractical for recreational boaters, except for equipment that can be soaked. Some barriers to implementation include the need for equipment that can consistently heat large volumes of water to high temperatures, heat loss due to environmental conditions and the type of surface being washed potentially reducing the temperature of water being applied^35^, and the elevated risk for personal injury or damage to equipment at temperatures > 50 °C. Since previous studies on the effect of hot water immersion as a decontamination method have shown that complete mortality occurs when organisms are exposed to water < 50 °C for longer periods than that of rinsing^27,29–31,41,42^, hot water application would nonetheless remain effective to decontaminate smaller equipment that can be immersed, or compartments that can be flooded, as opposed to rinsing where the exposure may be too brief, as shown with our tested durations of 2 s to 10 s.

Our experiments on air-drying provide some support for the recommended durations of two to seven days. Overall, we observed that increasing air-drying duration increases mortality and reduces viability among all aquatic invasive species, in line with previous studies. Air-drying for three days was sufficient for complete mortality among smaller invertebrates such as zebra mussels and spiny waterfleas, and one week of air-drying could ensure that plant fragments are non-viable, despite the minimum duration differing among the plant species that we tested. Tolerance to desiccation was especially notable among banded mystery snails which had low mortality for our tested durations, indicating that adults of this species of operculate snails can withstand being out of water for extended periods. The literature shows that aquatic invasive snail survival following air-drying varies considerably among species; for instance, complete mortality occurred among New Zealand mudsnails after 48 h of air-drying^28,43^, whereas others such as bladder snails, channeled apple snails, Chinese mystery snails, and island apple snails can survive from 14 to 154 days out of water^12,28,40,44^. Similarly, while the macrophytes in our study were not viable after one week of air-drying and species such as least duckweed, water fern, and Canadian waterweed had at least 90% mortality within one to five hours^45^, others such as parrot’s feather and New Zealand pygmyweed are hardier, requiring approximately 9 to 23 days of air-drying for the same mortality rate^27^. Air temperature or relative humidity were not included in the analyses for our study, since all replicates for the air-drying experiments were conducted within the same week, with the start of trials for each air-drying duration staggered such that the end of each exposure period coincided on the last day. However, based on the evidence from the literature, recreational boaters should be mindful of equipment and compartments such as live wells, ballast tanks, the bilge and engine, that may retain humidity or pooled water, and which would enable any organisms present to survive for longer periods^5,8,24,46^. Overall, despite air-drying seemingly being the easiest means of decontamination to implement, its efficacy cannot be generalised to include all invasive gastropods or plants, and recreational boaters should be aware of the type of AIS potentially present in the lakes they visit to ensure that the appropriate decontamination measures are applied. A limitation of our analyses for both the air-drying and the hot water experiments is that complete mortality was often not achieved with the air-drying durations and water temperatures tested. The experiments could be improved by including more intermediate treatment groups, for instance the addition of 60 h, 72 h, and 96 h air-drying for all species, and temperatures ranging from 15 to 70 °C in increments of 5 °C.

We previously reported that several studies found a difference in air-drying resistance based on the size or life stage of individuals of the same bivalve and gastropod species^24^; these support our observations on zebra mussels, where 100% mortality occurred after a shorter air-drying duration among smaller specimens (16 h) compared to larger ones (58 h). Combined with our spiny waterflea results, air-drying appears to be effective in killing smaller organisms or younger individuals of certain species, which might otherwise easily escape visual detection. However, larger individuals or organisms appear to be more tolerant of air-drying, and thus manual removal of larger, visible organisms and entangled plant material is recommended before implementing other decontamination techniques.

One aim of our study was to address the scarcity of experiments on the effect of sequentially applying more than one decontamination method. Although we only tested the efficacy of brief hot water exposure followed by air-drying, we determined that this combination was generally more effective than either method alone. By combining techniques, not only was a shorter air-drying duration generally required to reduce invertebrate survival or plant viability, but lower and more practical temperatures of 40 °C and 50 °C were also sufficient to contribute to complete mortality when coupled with air-drying among most species assessed, compared to the minimum of 60 °C when using hot water alone. To our knowledge, our study is the first to provide empirical evidence that combining decontamination methods improves efficacy, which can be especially useful against resilient species such as banded mystery snails. The latter could tolerate temperatures up to 65 °C or had a mortality rate of less than 50% after 6 days of air-drying only. However, mortality rates of up to 90% mortality can be achieved if snails are first exposed to 60 °C water, followed by 4 days of air-drying. Hence, combining lower water temperatures and shorter air-drying durations for the same effect could be a more feasible option for recreational boaters, potentially reducing the need for specialised equipment and time constraints. Although manually removing material attached to surfaces and draining residual water would eliminate some organisms present, the combination approach we described could also ensure that more species and individual organisms are killed prior to transport, as fouled watercraft and equipment potentially carry multiple species^5,8,20^ differing in resistance to specific decontamination methods. Future studies could empirically assess the efficacy of other combinations of decontamination methods and the minimum threshold for complete mortality, but it remains important to consider the ease of application, effort, and safety for recreational boaters. Our study has shown that decontamination methods as currently recommended for recreational boaters may not be optimal at targeting the diversity of AIS potentially transported via recreational watercraft. Hence, current recreational watercraft decontamination guidelines may need amendments to encourage the implementation of either the most effective treatment conditions, or a series of decontamination methods to help prevent the secondary spread of AIS by reducing viable propagules transported overland.

## Methods

All experiments were conducted at the Queen’s University Biological Station (QUBS; 44.568 N, 76.325 W) from May 22 to August 23, 2019, and at the Queen’s University campus (44.225 N, 76.495 W) from October 10 to 17, 2019, in Ontario, Canada.

### Pressure-washing experiments

We tested the efficacy of five water pressures (50, 125, 550, 900, 1950 psi) plus a control (0 psi) to remove a known amount of material from surfaces. We used a garden hose fitted with a spray nozzle to produce the 50 psi pressure, and for higher pressures, two commercially available electric pressure-washers (Sun Joe SPX3000 and Sun Joe SPX4600) fitted with 25° spray tips (Supplementary Figure S10), recommended for gentle lifting and cleaning. All equipment was connected to an outdoors faucet. The surfaces to be washed were 20 cm by 20 cm aluminum tiles secured to a metal frame oriented both perpendicular to the ground, and at a 20° angle to imitate the deadrise angle of boat hulls. During pressure-washing, the nozzle was held at approximately 30 cm from the surface to be washed, with the spray applied in a sideways unidirectional motion, covering an area of 75 cm by 60 cm that included the tiles, in 15 s. Tiles in the 0 psi control group were handled and secured to the frame for the same amount of time as in treatment groups, but did not undergo any washing. Each treatment (pressure by orientation) was replicated thrice.

The first experiment used tiles naturally colonized with periphyton that established over a 3-week period by suspending the tiles from wooden frames at approximately 1 m below the surface of Lake Opinicon (44.559 N, 76.327 W; Supplementary Figure S11). We expected that the periphyton would enable small particles such as seeds, plankton, eggs and larvae to adhere to the surface when removed from the water column. Upon retrieval, the periphyton from twelve random tiles was immediately scraped for later analysis (positive control group). The remaining tiles were randomly allocated to each treatment or control group, with equal numbers on the vertical (n = 3 per treatment) and angled (n = 3) sides of the washing frame. After pressure-washing, the residual periphyton attached to each tile was scraped with a toothbrush and collected in 75 mL to 100 mL of water, filtered using pre-weighed Whatman Grade 1 qualitative filter papers, and dried at 60 °C for at least 2 h until a constant mass was achieved. The dry mass of periphyton and residue per tile, for each treatment group and orientation, was calculated and recorded as the outcome measure for the periphyton experiments.

In the second experiment, 15 individual leaves of Eurasian watermilfoil (*Myriophyllum spicatum*) were randomly stuck to each of 12 aluminum tiles per treatment group, using 15 ml of extra-strong water-soluble hair gel (Garnier Fructis Extra Strong Gel 600 g) spread uniformly over the surface of the tile, similar to Rothlisberger et al.^20^. This experiment allowed us to control the amount of material present before and after washing, while the water-soluble gel facilitated the attachment of particles to surfaces. Six tiles were randomly allocated to each of the vertical and angled sides of the frame, and we recorded the number of whole leaflets and fragments remaining after washing and handling. Each treatment was replicated four times.

### Effects of hot water and/or air-drying on survival or viability

These experiments were conducted using three species of invertebrates (banded mystery snails, *Viviparus georgianus*; zebra mussels, *Dreissena polymorpha*; and spiny waterfleas, *Bythotrephes cederstroemi*) and three species of aquatic plants (Eurasian watermilfoil, *Myriophyllum spicatum*; Carolina fanwort, *Cabomba caroliniana*; and European frogbit, *Hydrocharis morsus-ranae*). We included ten healthy individuals (invertebrates) or ten 10 cm-long fragments (plants) per treatment, replicated three times, except for European frogbit, where a whole rosette was used per group, with four replicates.

Banded mystery snails and zebra mussels were manually collected from rocks and sediment at Lake Opinicon. Medium-sized adult snails (15-20 mm) and zebra mussels of two size classes (small: 8–12 mm, and large: 15–20 mm) were selected. All specimens were kept in flow-through tanks with a direct supply of filtered lake water for five to seven days prior to experiments, and we included only healthy individuals in the trials. Spiny waterfleas collected from Lake Ontario (44.221 N, 76.502 W) with a 50-µm tow net were used in the experiments on the same day as collection. Eurasian watermilfoil was collected from Lake Opinicon using a sampling rake, and Carolina fanwort was manually pulled from a headwater stream (44.526 N, 77.898 W) and transported to QUBS in plastic bags within sealed coolers to prevent desiccation and accidental release. We cut 10-cm fragments along each strand, at least 30 cm away from the roots and apices, and counted the number of leaves from each fragment to determine the total per group prior to treatment. None of the fragments had any roots or shoots at this time. European frogbit was collected from roadside ponds near QUBS. Any stolon or root was trimmed to a length of 5 cm when separating the rosettes. We recorded the number of leaves, roots, stolons, fruits, flowers, and turions per rosette before and after treatment.

After each trial, the plant and invertebrate specimens were placed in individual, labelled compartments of clear tackle boxes (or, for spiny waterfleas, tubes fitted with 50 µm mesh at both ends) that were immersed in tanks or containers of filtered lake water for monitoring. We recorded the number of survivors among banded mystery snails and zebra mussels after 24 h, and among spiny waterfleas after 4 h. Snails were classified as alive if they retracted their body into the shells when stimulated. Inactive specimens were considered alive if (1) the body retracted further when the operculum was tapped, or (2) there was resistance when the operculum was gently tugged with tweezers. Actively filter-feeding zebra mussels were marked as alive, whereas those that did not retract their siphon or close their valves when touched with a probe were considered dead. If the valves were initially closed, the mussels were deemed to be alive if they reopened during a ten-minute observation period. Spiny waterfleas were considered alive if they were actively swimming after immersion. Inactive individuals were transferred to a Petri dish and observed under a dissecting scope, and the absence of any internal movement confirmed that the specimen was dead.

The tanks for the plant fragments were fitted with two air-bubblers, and we added top and side sources of light, so that the fragments received 12 h of light daily. The position of the boxes was rotated daily by moving the bottom-most box to the top, to allow for even exposure to light, and the water was changed weekly. We monitored the fragments over a period of 28 days for all experiments (21 days for the acclimation experiment), recording the number of leaves, roots and lateral shoots (side branches) weekly. Although we recorded the number of all European frogbit structures present before and after the tests, we included only the number of new leaves and new turions in the final analyses.

#### Hot water exposure only

We used five water temperatures (25 °C, 40 °C, 50 °C, 60 °C, and 70 °C) and three immersion times simulating rinsing through brief exposure (2 s, 5 s, and 10 s) for all species, except spiny waterfleas where only 25 °C, 40 °C and 50 °C were used due to complete mortality at higher temperatures during screening trials. All specimens were placed in mesh containers that were lowered in a water bath held at the tested water temperature. The chosen durations of 2 s to 10 s were intended to simulate brief exposure to hot water during rinsing, where exposure duration is limited due to water running off surfaces. We did not assess the temperature at the point of contact as it would vary depending on environmental conditions and cannot be controlled or monitored by the user.

We also performed acclimation experiments to determine if AIS survival after hot water exposure was affected by the temperature to which the specimens were initially acclimated. Here, we included only banded mystery snails, large zebra mussels, and Eurasian watermilfoil. Specimens were placed in tanks of filtered lake water which were gradually brought to 15 °C, 20 °C (inside incubators), 25 °C and 30 °C (using aquarium water heaters) at rates of approximately 1.5 °C to 2 °C per 24 h. The specimens were then allowed to acclimate to the target temperature for seven days. Due to elevated zebra mussel and banded mystery snail mortality during acclimation at 30 °C, the invertebrates from this group were not included in subsequent trials. Otherwise, specimens from each acclimation group were immersed in water at 25 °C, 40 °C, 50 °C, 60 °C, or 70 °C for five seconds as previously described.

#### Air-drying only

Groups of test organisms (except spiny waterfleas) were allowed to air-dry outdoors, away from direct sunlight and rain inside a screen tent at QUBS, for 1 h, 3 h, 12 h, 1.5 days, 2.5 days, 5 days, and 7 days. We also included additional air-drying durations of 3 and 4 days for banded mystery snails after observing a sharp decline in the number of survivors between 2.5 and 5 days. Trials with spiny waterfleas were performed in the laboratory at Queen’s University, for durations of 1 h and 3 h, due to complete mortality after air-drying for 12 h.

#### Hot water and air-drying combination

To assess the efficacy of sequentially applying two decontamination methods, we immersed the test specimens in hot water (25 °C, 40 °C, 50 °C, 60 °C, and 70 °C) for 5 s before immediately allowing them to air-dry outdoors for 3 h, 12 h, 1.5 days, 2.5 days, and 5 days. In addition, snails were subjected to 4 days of air-drying after hot water exposure, and spiny waterfleas were only exposed to water at 25 °C, 40 °C, and 50 °C, combined with air-drying durations of 1 h, 3 h, and 12 h due to complete mortality at higher water temperatures and longer air-drying durations.

### Statistical analyses

We applied generalised linear models (GLMs) to analyse the data from all experiments, except those for spiny waterfleas where we used Analyses of Variance (ANOVA) with predictor variables as factors, as there were fewer treatment groups. We determined the best data distribution for all experiments by examining the diagnostic plots residuals, and the best models using Aikaike’s Information Criteria (AIC) values, or quasi-AIC (qAIC) if there was significant dispersion in the data. All analyses were performed using the statistical software R (version R-3.5.2, R Core Team 2020), and figures were created with the ggplot2 package (Wickham 2016). Model comparison and selection are shown in Supplementary Tables S11 to S17.

From the pressure washing experiments, we estimated the pressure output required to remove the most periphyton or leaf fragments from surfaces using the best fit regression models for the periphyton and gel experiments respectively. We did not include orientation in the final models for the periphyton experiment as it did not have a statistically significant effect on the amount of periphyton residue.

To determine the relationship between treatment conditions and survival among banded mystery snails and zebra mussels, we analysed the data using GLMs for a binomial distribution (logistic regression). We then used the best fitted regressions models to estimate the conditions that produced 50%, 90% and 99% mortality. The two-way ANOVAs on spiny waterfleas tested both the main effects and interaction of predictor variables. A significance level of 0.05 was used for all tests, and we conducted post-hoc tests (Tukey HSD) to evaluate the difference among levels if a significant interaction or main effects was detected.

We used Poisson regressions to determine the relationship between treatment conditions and growth among plants, post-treatment. We assigned a unique number (“plant ID”) to each group of ten Eurasian watermilfoil and Carolina fanwort fragments, or whole European frogbit rosette, and the analyses were repeated for each plant structure of interest (leaves, roots, branches, and turions). Growth, the response variable used in all analyses, was the difference between the number of a given structure recorded during the last week of monitoring and the minimum count recorded at any week for each unique plant ID, as a quantitative indicator of new growth at the end of the recovery period. The data were transformed by adding 1 to the count difference to avoid zero values, and thus meet the assumptions of Poisson regressions. Finally, we used the best fit regression models to estimate treatment conditions that produced counts of less than one as a measure of no new growth at the end of the recovery period.

### Use of plants

All local (Ontario, Canada) guidelines and legislation were adhered to for the use of plants in this study. No species at risk of extinction were collected or harmed in conducting this study, in accordance with the IUCN Policy Statement on Research Involving Species at Risk of Extinction. No permits or licenses were required for the collection and handling of the aquatic invasive plant species used in this study, as per the Ontario Ministry of Natural Resources and Forestry (OMNRF). Eurasian watermilfoil (*Myriophyllum spicatum*) samples were taken from Lake Opinicon, accessed through the Queen’s University Biological Station property, hence requiring no permissions. No voucher specimens of the plants used were kept when the study was conducted; all plant identification was performed by SM based on publicly available reference guides from the OMNRF, and in consultation with OMNRF invasive species biologists.

## Supplementary Information

Supplementary Figures.

Supplementary Tables.

## Data Availability

The datasets used and/or analysed during the current study is available from the Queen’s University Biological Station Data Archive Dataverse at https://doi.org/10.5683/SP3/33EBNJ .

## References

[1] Drake, D. A. R., Bailey, S. A. & Mandrak, N. E. Ecological risk assessment of recreational boating as a pathway for the secondary spread of aquatic invasive species in the Great Lakes Basin. *DFO Canadian Science Advisory Secretariat* v + 85 (2017).

[2] Ricciardi A (2007). Are modern biological invasions an unprecedented form of global change?. Conserv. Biol..

[3] Van der Zanden MJ, Olden JD (2008). A management framework for preventing the secondary spread of aquatic invasive species. Can. J. Fish Aquat. Sci..

[4] De Ventura L, Weissert N, Tobias R, Kopp K, Jokela J (2016). Overland transport of recreational boats as a spreading vector of zebra mussel *Dreissena polymorpha*. Biol. Invasions.

[5] Johnson LE, Ricciardi A, Carlton JT (2001). Overland dispersal of aquatic invasive species: A risk assessment of transient recreational boating. Ecol. Appl..

[6] Leung B, Bossenbroek JM, Lodge DM (2006). Boats, pathways, and aquatic biological invasions: Estimating dispersal potential with gravity models. Biol. Invasions.

[7] Bacela-Spychalska K, Grabowski M, Rewicz T, Konopacka A, Wattier R (2013). The, “killer shrimp’* Dikerogammarus villosu*s (Crustacea, Amphipoda) invading Alpine lakes: Overland transport by recreational boats and scuba-diving gear as potential entry vectors?. Aquat. Conserv..

[8] Kelly NE, Wantola K, Weisz E, Yan ND (2013). Recreational boats as a vector of secondary spread for aquatic invasive species and native crustacean zooplankton. Biol. Invasions.

[9] Kerfoot WC (2016). A plague of waterfleas (*Bythotrephes*): Impacts on microcrustacean community structure, seasonal biomass, and secondary production in a large inland-lake complex. Biol. Invasions.

[10] Alonso A, Valle-Torres G, Castro-Diez P (2016). Survival of an invasive aquatic snail to overland translocation in non-aquatic media: Implications for spreading. Limnologica.

[11] Collas FPL, Karatayev AY, Burlakova LE, Leuven RSEW (2018). Detachment rates of dreissenid mussels after boat hull-mediated overland dispersal. Hydrobiologia.

[12] Havel JE (2011). Survival of the exotic Chinese mystery snail (*Cipangopaludina chinensis malleata*) during air exposure and implications for overland dispersal by boats. Hydrobiologia.

[13] Ricciardi A, Serrouya R, Whoriskey FG (1995). Aerial exposure tolerance of zebra and quagga mussels (Bivalvia: Dreissenidae): Implications for overland dispersal. Can. J. Fish. Aquat. Sci..

[14] Bailey SA (2004). Salinity tolerance of diapausing eggs of freshwater zooplankton. Freshw. Biol..

[15] Gaff DF, Oliver M (2013). The evolution of desiccation tolerance in angiosperm plants: A rare yet common phenomenon. Funct. Plant Biol..

[16] Ricciardi A, Rasmussen JB (1998). Predicting the identity and impact of future biological invaders: A priority for aquatic resource management. Can. J. Fish Aquat. Sci..

[17] Chivers C, Leung B (2012). Predicting invasions: Alternative models of human-mediated dispersal and interactions between dispersal network structure and Allee effects. J. Appl. Ecol..

[18] Drake, D. A. R. Overland spread of aquatic invasive species among freshwater ecosystems due to recreational boating in canada. *DFO Canadian Science Advisory Secretariat*, vi + 38 (2017).

[19] Hunt LM, Morris DM, Drake DAR, Buckley JD, Johnson TB (2019). Predicting spatial patterns of recreational boating to understand potential impacts to fisheries and aquatic ecosystems. Fish. Res..

[20] Rothlisberger JD, Chadderton WL, McNulty J, Lodge DM (2010). Aquatic invasive species transport via trailered boats: What is being moved, who is moving it, and what can be done. Fisheries.

[21] Blackburn TM, Lockwood JL, Cassey P (2015). The influence of numbers on invasion success. Mol. Ecol..

[22] Sinclair JS, Arnott SE (2016). Strength in size not numbers: Propagule size more important than number in sexually reproducing populations. Biol. Invasions.

[23] Sinclair JS, Arnott SE (2017). Relative importance of colonist quantity, quality, and arrival frequency to the extinction of two zooplankton species. Oecologia.

[24] Mohit S, Johnson TB, Arnott SE (2021). Recreational watercraft decontamination: Can current recommendations reduce aquatic invasive species spread?. Manag. Biol. Invasions.

[25] Canadian Council on Invasive Species. *Clean Drain Dry*, https://canadainvasives.ca/programs/clean-drain-dry/ (2021).

[26] Ontario Ministry of Natural Resources and Forestry. *Boater Action Plan*, https://files.ontario.ca/boater_action_plan_invasivespecies-2017.pdf (2017).

[27] Anderson L, Dunn A, Rosewarne P, Stebbing P (2015). Invaders in hot water: A simple decontamination method to prevent the accidental spread of aquatic invasive non-native species. Biol. Invasions.

[28] Collas FPL (2014). Effects of desiccation on native and non-native molluscs in rivers. Freshw. Biol..

[29] Beyer J, Moy P, De Stasio B (2011). Acute upper thermal limits of three aquatic invasive invertebrates: Hot water treatment to prevent upstream transport of invasive species. Environ. Manag..

[30] Coughlan NE (2019). Better biosecurity: Spread-prevention of the invasive Asian clam, *Corbicula fluminea* (Muller, 1774). Manag. Biol. Invasions.

[31] Shannon C, Quinn CH, Stebbing PD, Hassall C, Dunn AM (2018). The practical application of hot water to reduce the introduction and spread of aquatic invasive alien species. Manag. Biol. Invasions.

[32] Comeau S (2011). Susceptibility of quagga mussels (*Dreissena rostriformis bugensis*) to hot-water sprays as a means of watercraft decontamination. Biofouling.

[33] Crane K (2019). Full steam ahead: Direct steam exposure to inhibit spread of invasive aquatic macrophytes. Biol. Invasions.

[34] Morse JT (2009). Assessing the effects of application time and temperature on the efficacy of hot-water sprays to mitigate fouling by *Dreissena polymorpha* (zebra mussels Pallas). Biofouling.

[35] Bradbeer SJ (2021). The effectiveness of hot water pressurized spray in field conditions to slow the spread of invasive alien species. Manag. Biol. Invasions.

[36] Wada T, Matsukura K (2011). Linkage of cold hardiness with desiccation tolerance in the invasive freshwater apple snail, *Pomacea canaliculata* (Caenogastropoda: Ampullariidae). J. Molluscan Stud..

[37] Atkinson J, King NG, Wilmes SB, Moore PJ (2020). Summer and winter marine heatwaves favor an invasive over native seaweeds. J. Phycol..

[38] Wong, W. H., Gerstenberger, S. & Watters, A. Using pressurized hot water spray to kill and remove dreissenid mussels on watercraft: Field testing on the efficacy of water temperature, high pressure, and duration of exposure, New York (2014).

[39] Tamburi NE, Seuffert ME, Martin PR (2018). Temperature-induced plasticity in morphology and relative shell weight in the invasive apple snail *Pomacea canaliculata*. J. Therm. Biol.

[40] Yoshida K, Matsukura K, Cazzaniga NJ, Wada T (2014). Tolerance to low temperature and desiccation in two invasive apple snails, *Pomacea canaliculata* and *P. maculata *(Caenogastropoda: Ampullariidae), collected in their original distribution area (northern and central Argentina). J. Molluscan Stud..

[41] De Stasio BT, Acy CN, Frankel KE, Fritz GM, Lawhun SD (2019). Tests of disinfection methods for invasive snails and zooplankton: Effects of treatment methods and contaminated materials. Lake Reserv. Manag..

[42] Sebire M, Rimmer G, Hicks R, Parker SJ, Stebbing PD (2018). A preliminary investigation into biosecurity treatments to manage the invasive killer shrimp (*Dikerogammarus villosus*). Manag. Biol. Invasions.

[43] Richards DC, O'Connell P, Shinn DC (2004). Simple control method to limit the spread of the New Zealand mudsnail *Potamopyrgus antipodarum*. North Am. J. Fish. Manag..

[44] Bernatis JL, McGaw IJ, Cross CL (2016). Abiotic tolerances in different life stages of apple snails *Pomacea canaliculata* and *Pomacea maculata* and the implications for distribution. J. Shellfish Res..

[45] Coughlan NE, Cuthbert RN, Kelly TC, Jansen MAK (2018). Parched plants: Survival and viability of invasive aquatic macrophytes following exposure to various desiccation regimes. Aquat. Bot..

[46] Campbell, T., Verboomen, T., Montz, G. & Seilheimer, T. Volume and contents of residual water in recreational watercraft ballast systems. *Manag. Biol. Invasions* **7**, 281–286. 10.3391/mbi.2016.7.3.07 (2016).

