# Supplementary material for: Watercraft decontamination practices to reduce the viability of aquatic invasive species implicated in overland transport
Source: Sci Rep. 2023 May 4;13:7238. doi: 10.1038/s41598-023-33204-0 (PMC10160014; doi:10.1038/s41598-023-33204-0)
Supplement: Supplementary file 1 — Supplementary Figures. [file 41598_2023_33204_MOESM1_ESM.docx]

**Supplementary Figures**

**Results from air-drying experiments on small zebra mussels**


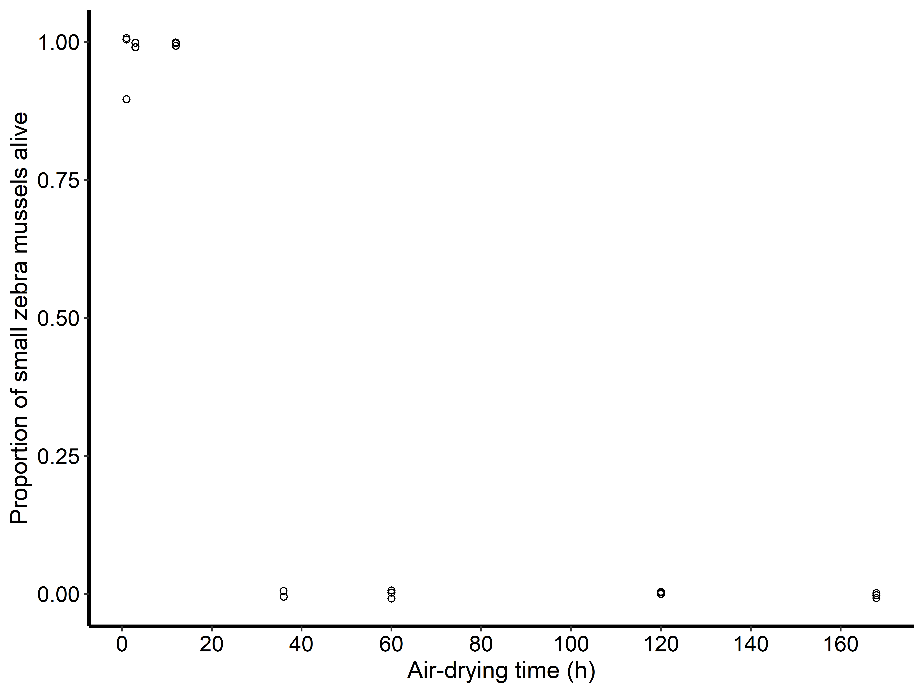


Figure S1. Scatterplot showing the proportion of small (8-12mm) zebra mussels surviving after air-drying for 1h to 7 days. Survival was very high in air-drying groups of up to 12h, with a sudden drop resulting in complete mortality in the air-drying groups of 36h and above.

**Results from hot water experiments on macrophytes**


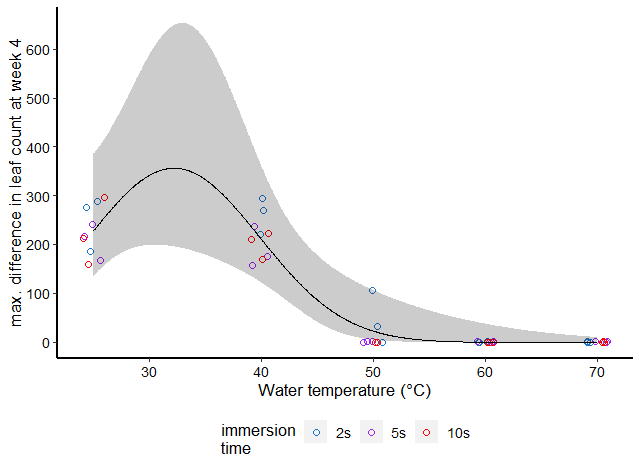

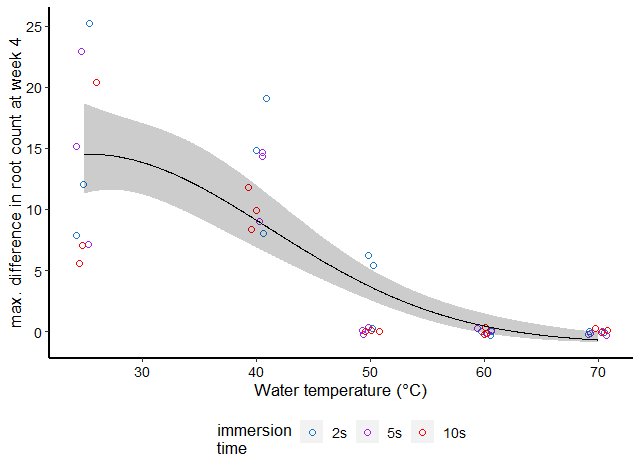

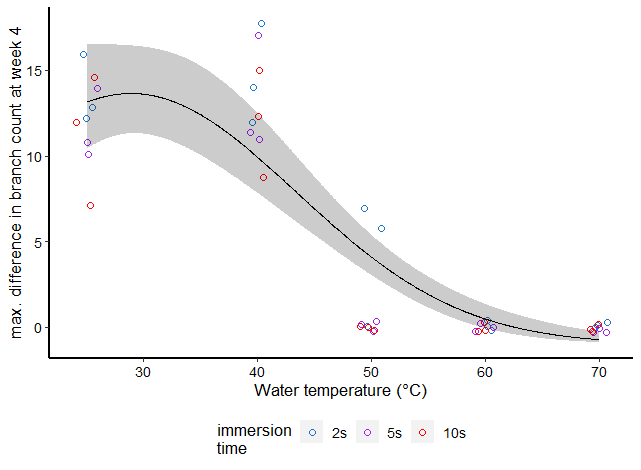


Figure S2. Relationship between new leaf (top left), root (top right) and branch (bottom) growth at week 4 and hot water temperature among Eurasian watermilfoil fragments. Jittered circles represent the observed data; the solid line and the shaded area represent the regression and the 95% confidence band, respectively.


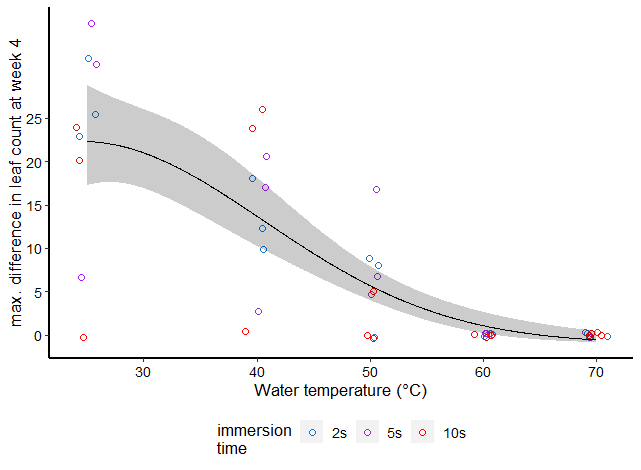

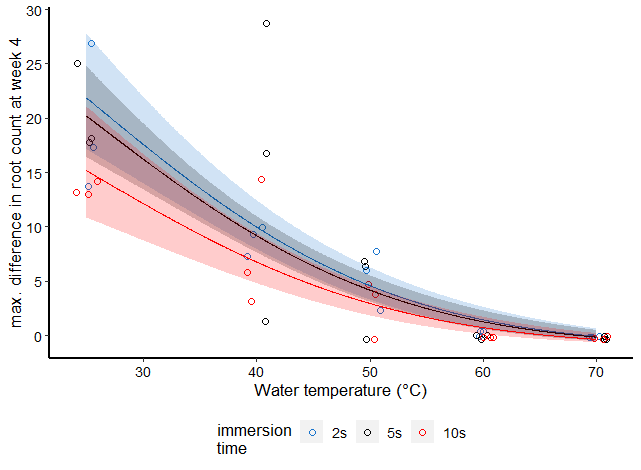

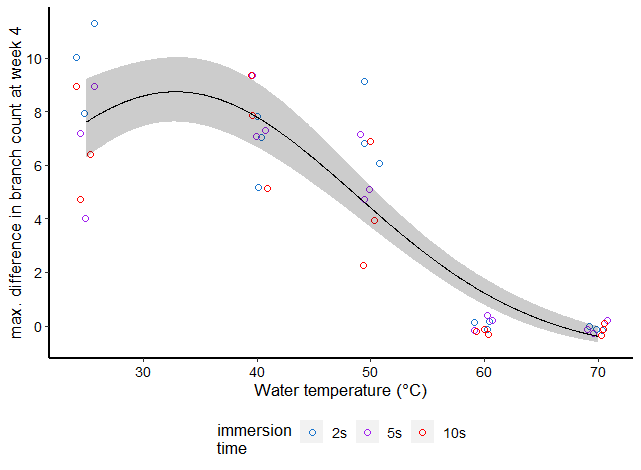


Figure S3. Relationship between new leaf (top left), root (top right) and branch (bottom) growth at week 4 and hot water temperature among Carolina fanwort fragments.


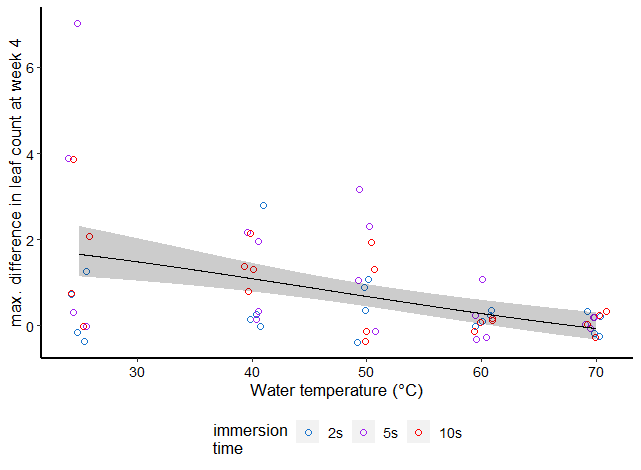

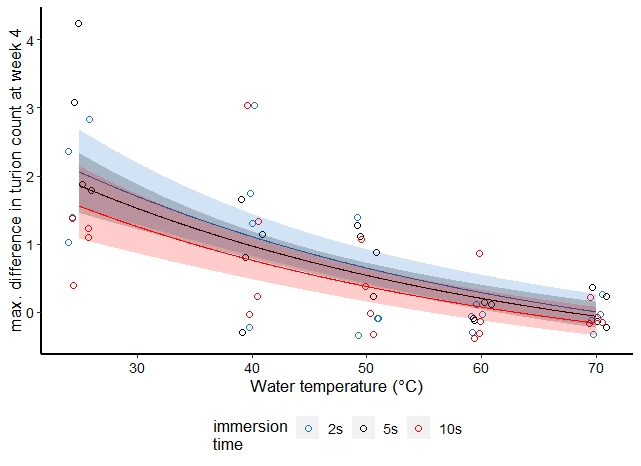


Figure S4. Relationship between hot water temperature and new leaf (left) and turion (right) growth at week 4 among European frogbit rosettes.

**Results from air-drying experiments on macrophytes**


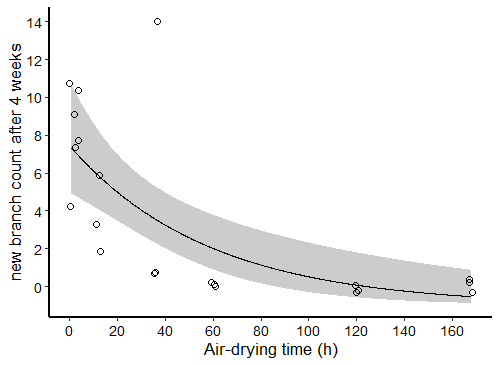

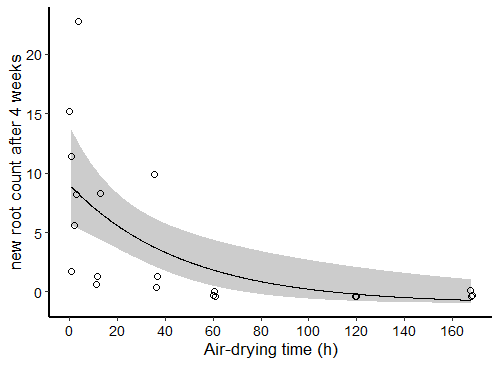

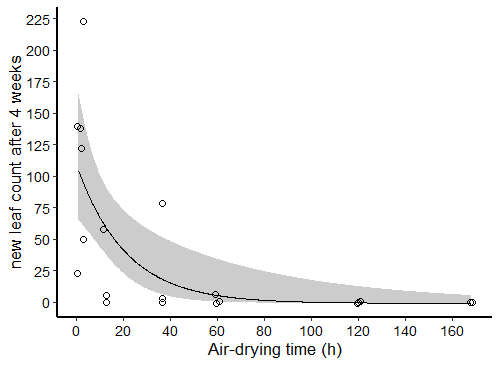


Figure S5. Relationship between air-drying duration and new leaf (top left), root (top right), and branch (bottom) growth among Eurasian watermilfoil.


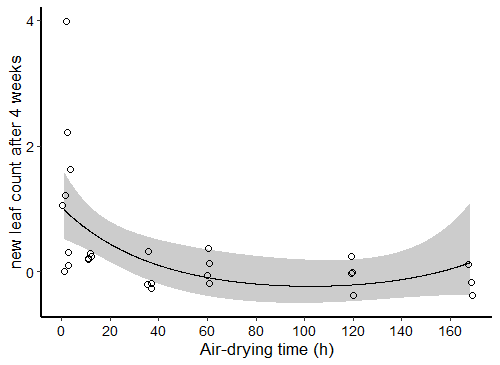

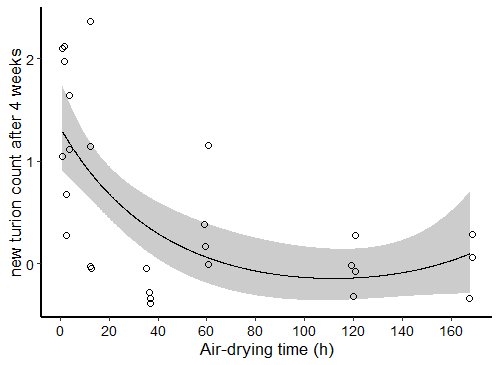


Figure S6. Relationship between air-drying duration and new leaf (left) and turion (right) growth among European frogbit.

**Results from experiments combining hot water and air-drying on macrophytes**


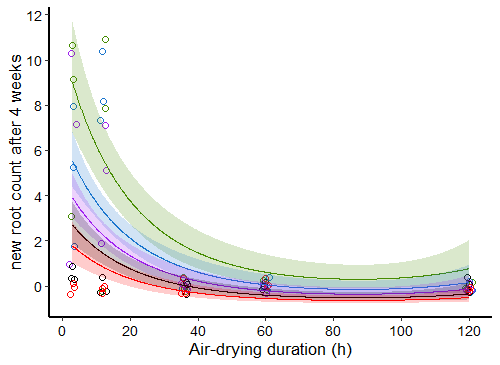

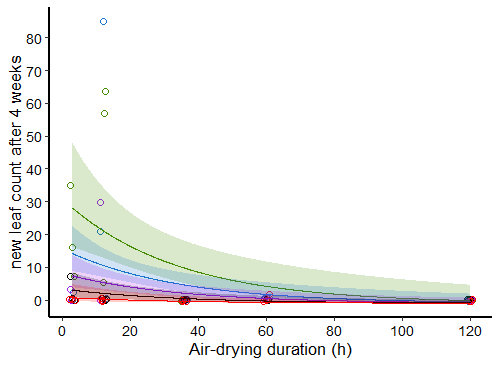

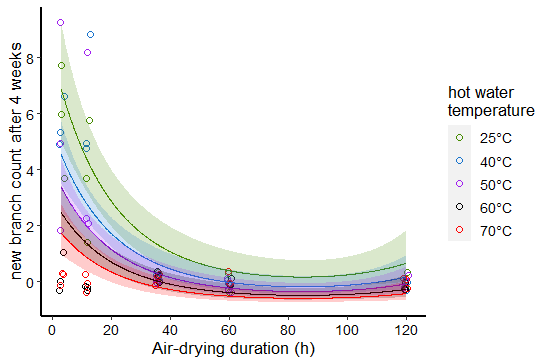


Figure S7. New leaf (top left), root (top right), and branch (bottom) growth after four weeks among Eurasian watermilfoil fragments with increasing air-drying duration, after exposure to hot water.


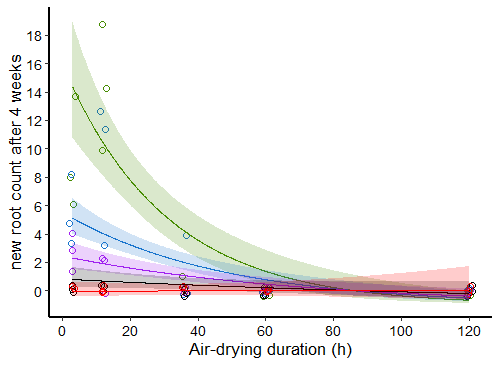

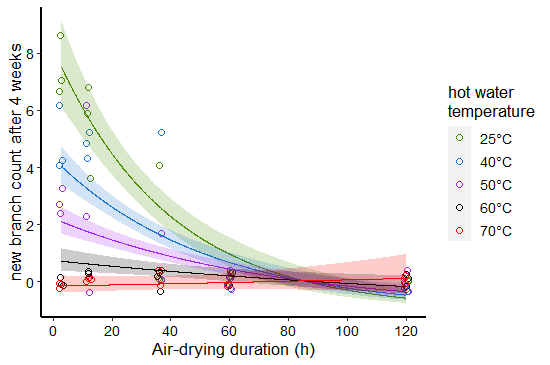

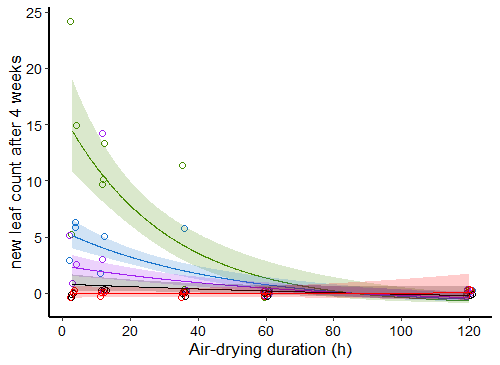


Figure S8. New leaf (top left), root (top right), and branch (bottom) growth after four weeks among Carolina fanwort fragments with increasing air-drying duration, after exposure to hot water.


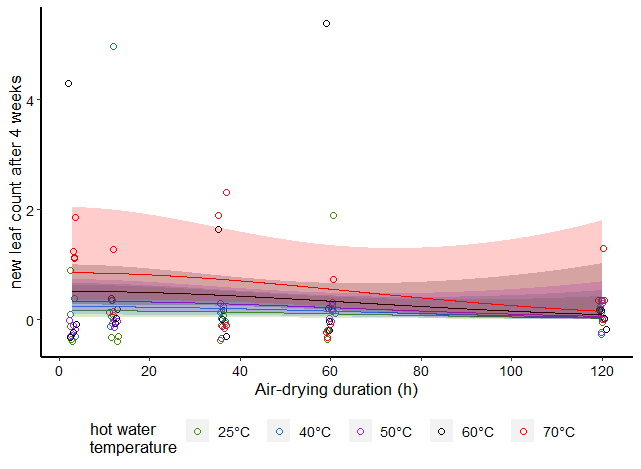

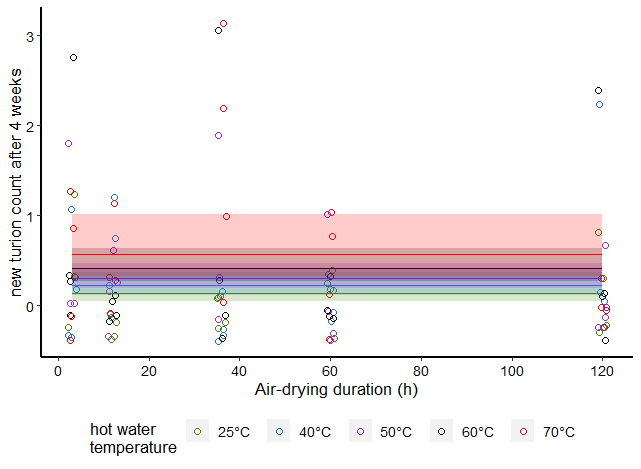


Figure S9. New leaf (left) and turion (right) growth at week 4 among European frogbit rosettes with increasing air-drying duration, after exposure to hot water.

**Pressure washer nozzle**


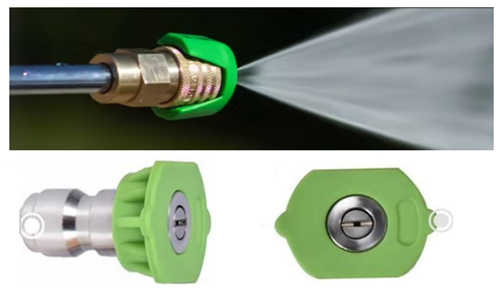


Source: snowjoe.com

Figure S10. Pressure washer nozzles with 25° spray pattern. Top row shows default nozzle from the manufacturer of the pressure washers used in the experiments. Bottom row shows additional nozzles purchased to generate other levels of pressure tested.

**Characteristics of Lake Opinicon**


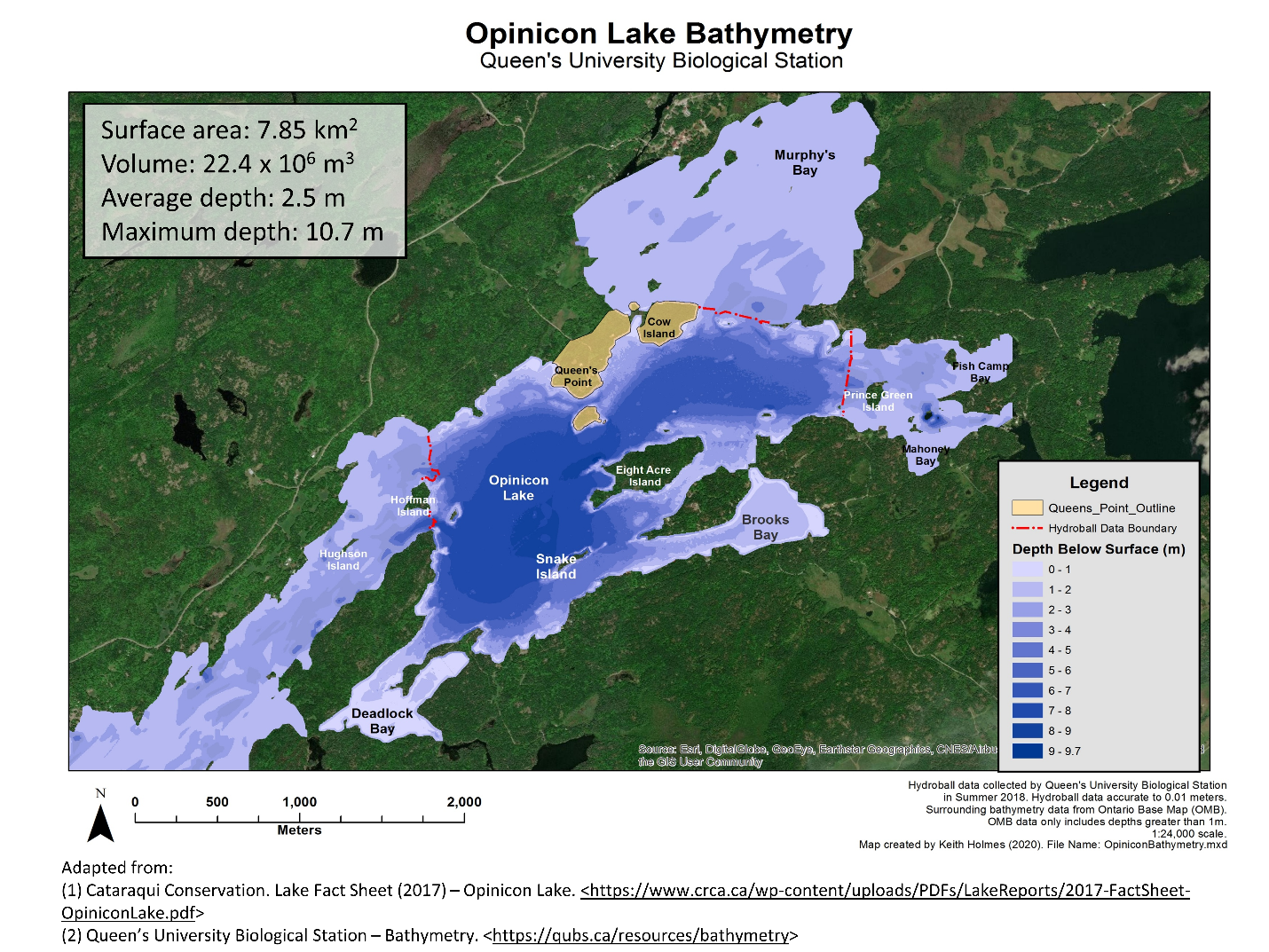


Figure S11. Bathymetry and properties of Lake Opinicon, showing study site (Queen’s Point). Adapted from Cataraqui Conservation, *Lake Fact Sheet* (2017) – Opinicon Lake (<https://www.crca.ca/wp-content/uploads/PDFs/LakeReports/2017-FactSheet-OpiniconLake.pdf>) and Queen’s University Biological Station, *Opinicon Lake bathymetry* (2020), created by Keith Holmes (<https://qubsfiles.s3-us-west-2.amazonaws.com/bathymetry/OpiniconBathymetry.jpg>).
