# Supplementary material for: Watercraft decontamination practices to reduce the viability of aquatic invasive species implicated in overland transport
Source: Sci Rep. 2023 May 4;13:7238. doi: 10.1038/s41598-023-33204-0 (PMC10160014; doi:10.1038/s41598-023-33204-0)
Supplement: Supplementary file 2 — Supplementary Tables. [file 41598_2023_33204_MOESM2_ESM.docx]

**Supplementary Tables**

**Pressure washing experiments**

| Table S1. Results from regression analyses for pressure washing experiments using surfaces naturally colonised by periphyton, or gel-seeded plant fragments. Best fit regression models are indicated. | | | |
| --- | --- | --- | --- |
| **Variable** | **Regression coefficient** | **Standard Error (SE)** | **p-value** |
| *Periphyton experiment (GLM; Gaussian regression)* | | | |
| Pressure^2^ | 1.811 x 10^-8^ | 4.785 x 10^-9^ | < 0.001* |
| Pressure | -3.339 x 10^-5^ | 9.545 x 10^-6^ | < 0.001* |
| *Gel experiment (GLM; quasi-Poisson regression)* | | | |
| Pressure^2^ | 1.526 x 10^-6^ | 1.934 x 10^-7^ | < 0.001* |
| Pressure | -3.398x10^-3^ | 3.786x10^-4^ | < 0.001* |
| Orientation (vertical relative to angled) | 0.306 | 0.103 | 0.003* |
| **Note:** * statistically significant | | | |

**Experiments on invertebrates**

*Hot water experiments*

| Table S2. Results from logistic regression analyses for brief hot water immersion experiments (simulating rinsing) on banded mystery snails, including acclimation experiment. Best fit regression models are indicated. | | | |
| --- | --- | --- | --- |
| **Variable** | **Estimate** | **95% CI** | **p-value** |
| *Hot water experiment (GLM; quasi-binomial regression)* | | | |
| Temperature | -0.692 | -0.871 – -0.564 | < 0.001* |
| Exposure time | -0.473 | -0.713 – -0.292 | < 0.001* |
| *Acclimation experiment (GLM; quasi-binomial regression)* | | | |
| **Variable** | **Estimate** | **95% CI** | **p-value** |
| Hot water temperature^2^ | -0.006 | -0.010 – -0.002 | 0.005* |
| Hot water temperature | 0.381 | 0.067 – 0.728 | 0.026* |
| Acclimation temperature | -0.046 | -0.187 – 0.089 | 0.504 |
| **Note:** * statistically significant | | | |

| Table S3. Results from logistic regression analyses for brief hot water immersion experiments on two size classes of zebra mussels, and acclimation experiments on large zebra mussels. Best fit regression models are indicated. | | | |
| --- | --- | --- | --- |
| *Small zebra mussels (8-12mm – GLM; quasi-binomial regression)* | | | |
| **Variable** | **Estimate** | **95% CI** | **p-value** |
| Temperature^2^ | -0.009 | -0.016 – -0.004 | 0.005* |
| Temperature | 0.556 | 0.131 – 1.042 | 0.022* |
| Exposure time | -0.110 | -0.260 – 0.034 | 0.148 |
| *Large zebra mussels (15-20mm – GLM; quasi-binomial regression)* | | | |
| **Variable** | **Estimate** | **95% CI** | **p-value** |
| Temperature^2^ | -0.008 | 0.987 – 0.995 | < 0.001* |
| Temperature | 0.549 | 1.308 – 2.419 | 0.001* |
| Exposure time | -0.149 | 0.771 – 0.958 | 0.010* |
| *Acclimation experiment (15-20mm zebra mussels only – GLM; quasi-binomial regression)* | | | |
| **Variable** | **Estimate** | **95% CI** | **p-value** |
| Hot water temperature^2^ | -0.024 | -0.030 – -0.019 | < 0.001* |
| Hot water temperature | 1.523 | 1.137 – 1.947 | < 0.001* |
| Acclimation temperature | 0.202 | 0.098 – 0.322 | < 0.001* |
| **Note:** * statistically significant | | | |

*Air-drying experiments*

| Table S4. Results from logistic regression analyses for air-drying experiments on invertebrates. Best fit regression models are indicated. | | | |
| --- | --- | --- | --- |
| *Banded mystery snails (GLM; quasi-binomial regression)* | | | |
| **Variable** | **Estimate** | **95% CI** | **p-value** |
| Time^2^ | 0.0002 | 8.330 x 10^-6^ – 0.0004 | 0.063 |
| Time | -0.051 | -0.009 – -0.018 | 0.011* |
| *Small zebra mussels (8-12mm – GLM; quasi-binomial regression)* | | | |
| **Variable** | **Estimate** | **95% CI** | **p-value** |
| Time^2^ | -0.546 | -0.807 – -0.180 | 0.979 |
| Time | 8.789 | 8.025 – 9.553 | 0.946 |
| *Large zebra mussels (15-20mm – GLM; quasi-binomial regression)* | | | |
| **Variable** | **Estimate** | **95% CI** | **p-value** |
| Time^2^ | 0.9983 | 0.9979 – 0.9986 | < 0.001* |
| **Note:** * statistically significant | | | |

*Experiments combining hot water and air-drying*

| Table S5. Results from logistic regression analyses for combined treatment experiments using brief hot water exposure followed by air-drying among invertebrates. Best fit regression models are indicated. | | | |
| --- | --- | --- | --- |
| *Banded mystery snails (GLM; quasi-binomial regression)* | | | |
| **Variable** | **Estimate** | **95% CI** | **p-value** |
| Water temperature^2^ | -4.110 | -7.244 – -1.103 | < 0.001* |
| Water temperature | 0.361 | 0.214 – 0.522 | < 0.001* |
| Air-drying duration | -0.026 | -0.034 – -0.018 | < 0.001* |
| *Small zebra mussels (8-12mm – GLM; quasi-binomial regression)* | | | |
| **Variable** | **Estimate** | **95% CI** | **p-value** |
| Air-drying duration^2^ | -0.008 | -0.013 – -0.004 | 0.006* |
| *Large zebra mussels (15-20mm – GLM; quasi-binomial regression)* | | | |
| **Variable** | **Estimate** | **95% CI** | **p-value** |
| Air-drying duration^2^ | -0.004 | -0.006 – -0.003 | < 0.001* |
| **Note:** * statistically significant | | | |

**Experiments on macrophytes**

*Hot water experiments*

| Table S6. Results from quasi-Poisson regression analyses for hot water experiments on Eurasian watermilfoil, including acclimation experiments | | | | |
| --- | --- | --- | --- | --- |
|  | **Variable** | **Regression coefficient** | **Standard Error (SE)** | **p-value** |
| Leaf growth | Temperature^2^ | -0.009 | 0.004 | 0.022* |
|  | Temperature | 0.553 | 0.248 | 0.031* |
| Root growth | Temperature^2^ | -0.002 | 0.001 | 0.003* |
|  | Temperature | 0.099 | 0.050 | 0.053 |
| Branch growth | Temperature^2^ | -0.002 | 0.001 | < 0.001* |
|  | Temperature | 0.137 | 0.044 | 0.003* |
| *Acclimation experiment* | | | | |
|  | **Variable** | **Regression coefficient** | **Standard Error (SE)** | **p-value** |
| Leaf growth | Water temperature^2^ | -0.004 | 0.0004 | < 0.001* |
|  | Water temperature | 0.265 | 0.038 | < 0.001* |
|  | Acclimation temperature | -0.030 | 0.011 | < 0.001* |
| Root growth | Water temperature^2^ | -0.002 | 0.0004 | < 0.001* |
|  | Water temperature | 0.118 | 0.033 | < 0.001* |
| Branch growth | Water temperature^2^ | -0.002 | 0.0003 | < 0.001* |
|  | Water temperature | 0.173 | 0.024 | < 0.001* |
| **Note:** * statistically significant | | | | |

| Table S7. Results from quasi-Poisson regression analyses for hot water experiments on Carolina fanwort. | | | | |
| --- | --- | --- | --- | --- |
|  | **Variable** | **Regression coefficient** | **Standard Error (SE)** | **p-value** |
| Leaf growth | Temperature^2^ | -0.002 | 0.001 | 0.006* |
|  | Temperature | 0.094 | 0.053 | 0.082 |
| Root growth | Temperature^2^ | -7.517 x 10^-4^ | -7.712 x 10^-5^ | < 0.001* |
|  | Immersion time^2^ | -0.004 | 0.002 | 0.078 |
| Branch growth | Temperature^2^ | -0.002 | 0.0003 | < 0.001* |
|  | Temperature | 0.131 | 0.026 | < 0.001 |
| **Note:** * statistically significant | | | | |

| Table S8. Results from quasi-Poisson regression analyses for hot water experiments on European frogbit. | | | | |
| --- | --- | --- | --- | --- |
|  | **Variable** | **Regression coefficient** | **Standard Error (SE)** | **p-value** |
| Leaf growth | Temperature^2^ | -2.439 x 10^-4^ | 5.416 x 10^-5^ | < 0.001* |
| Turion growth | Temperature | -0.025 | 0.003 | < 0.001* |
|  | Immersion time | -0.022 | 0.016 | 0.158 |
| **Note:** * statistically significant | | | | |

*Air-drying experiments*

| Table S9. Results from Poisson/quasi-Poisson regression analyses for air-drying experiments on three aquatic plant species | | | | |
| --- | --- | --- | --- | --- |
|  | **Variable** | **Regression coefficient** | **Standard Error (SE)** | **p-value** |
| *Eurasian watermilfoil* | | | | |
| Leaf growth | Air-drying duration | -0.048 | 0.016 | 0.009* |
| Root growth | Air-drying duration | -0.021 | 0.006 | 0.004* |
| Branch growth | Air-drying duration | -0.017 | 0.005 | 0.002* |
| *Carolina fanwort* | | | | |
| Leaf growth | Air-drying duration | -0.018 | 0.008 | 0.030* |
| Root growth | Air-drying duration^2^ | -1.097 x 10^-4^ | 3.897 x 10^-5^ | 0.011* |
| Branch growth | Air-drying duration^2^ | -9.755 x 10^-5^ | 1.942 x 10^-5^ | < 0.001* |
| *European frogbit* | | | | |
| Leaf growth | Air-drying duration^2^ | 9.234 x 10^-5^ | 4.314 x 10^-5^ | 0.043* |
|  | Air-drying duration | -0.019 | -0.007 | 0.012* |
| Turion growth | Air-drying duration^2^ | 7.963 x 10^-5^ | 2.990 x 10^-5^ | 0.014* |
|  | Air-drying duration | -0.018 | -0.005 | < 0.001* |
| **Note:** * statistically significant | | | | |

*Experiments combining hot water and air-drying*

| Table S10. Results from Poisson/quasi-Poisson regression analyses for combination experiments on three aquatic plant species | | | | |
| --- | --- | --- | --- | --- |
|  | **Variable** | **Regression coefficient** | **Standard Error (SE)** | **p-value** |
| *Eurasian watermilfoil* | | | | |
| Leaf growth | Air-drying duration | -0.030 | 0.009 | 0.001* |
|  | Water temperature^2^ | -0.0007 | 0.0002 | < 0.001* |
| Root growth | Air-drying duration^2^ | 2.869 x 10^-4^ | 6.642 x 10^-5^ | < 0.001* |
|  | Air-drying duration | -0.050 | 0.008 | < 0.001* |
|  | Water temperature | -0.028 | 0.005 | < 0.001* |
| Branch growth | Air-drying duration^2^ | 2.864 x 10^-4^ | 6.885 x 10^-5^ | < 0.001* |
|  | Air-drying duration | -0.049 | 0.008 | < 0.001* |
|  | Water temperature | -0.024 | 0.005 | < 0.001* |
| *Carolina fanwort* | | | | |
| Leaf growth | Air-drying duration | -0.051 | 0.009 | < 0.001* |
|  | Hot water temperature | -0.064 | 0.008 | < 0.001* |
|  | Interaction | 0.0007 | 0.0002 | < 0.001* |
| Root growth | Air-drying duration | -0.039 | 0.005 | < 0.001* |
|  | Hot water temperature^2^ | -7.435 x 10^-4^ | 7.765 x 10^-5^ | < 0.001* |
|  | Interaction | 9.063 x 10^-6^ | 1.645 x 10^-6^ | < 0.001* |
| Branch growth | Air-drying duration | -0.030 | 0.003 | < 0.001* |
|  | Hot water temperature^2^ | -5.586 x 10^-4^ | 5.454 x 10^-5^ | < 0.001* |
|  | Interaction | 6.564 x 10^-6^ | 1.145 x 10^-6^ | < 0.001* |
| *European frogbit* | | | | |
| Leaf growth | Air-drying duration^2^ | -1.204 x 10^-4^ | 9.392 x 10^-5^ | 0.203 |
|  | Hot water temperature^2^ | 3.862 x 10^-4^ | 2.085 x 10^-4^ | 0.067 |
| Turion growth | Hot water temperature | 0.032 | 0.015 | 0.033* |
| **Note:** * statistically significant | | | | |

**Model selection based on AIC or qAIC for all experiments**

| Table S11. Comparison of generalised linear models for pressure experiments. | | |
| --- | --- | --- |
| **Periphyton experiment (Gaussian distribution)** | | **AIC** |
| A1 | algae dry mass = *a* + *b_1_*(pressure) | -535.85 |
| A2 | algae dry mass = *a* + *b_1_*(pressure^2^) + *b_2_*(pressure) | **-547.66** |
| A3 | algae dry mass = *a* + *b_1_*(pressure^2^) | -537.75 |
| A4 | algae dry mass = *a* + *b_1_*(pressure) + *b_2_*(orientation) | -537.09 |
| A5 | algae dry mass = *a* + *b_1_*(pressure) + *b_2_*(orientation) + *b_3_*(pressure:orientation) | -535.10 |
| **Gel experiment (Poisson distribution)** | | **qAIC** |
| G1 | log number of leaves = *a* + *b_1_*(pressure) | 479.00 |
| G2 | log number of leaves = *a* + *b_1_*(pressure^2^) | 503.34 |
| G3 | log number of leaves = *a* + *b_1_*(pressure^2^) + *b_2_*(pressure) | 419.46 |
| G5 | log number of leaves = *a* + *b_1_*(orientation) | 509.05 |
| G6 | log number of leaves = *a* + *b_1_*(pressure) + *b_2_*(orientation) + *b_3_*(pressure:orientation) | 472.62 |
| G7 | log number of leaves = *a* + *b_1_*(pressure) + *b_2_*(orientation) | 473.88 |
| G8 | log number of leaves = *a* + *b_1_*(pressure^2^) + *b_2_*(pressure) + *b_3_*(orientation) | **414.34** |
| Note: Models are shown with the link function applied to the response variable; *a* and *b_i_* represent regression coefficients; “:” represents the interaction between variables. Selected models based on AIC/qAIC and parsimony are indicated in boldface. | | |

| Table S12. Comparison of logistic regression models for experiments on banded mystery snails and zebra mussels, using qAIC. | | | | |
| --- | --- | --- | --- | --- |
|  | | Banded mystery snails | Small zebra mussels | Large zebra mussels |
| **Hot water experiment** | | **qAIC** | | |
| HW1 | log odds survival = *a* + *b_1_*(temperature) | 248.77 | 64.18 | 90.62 |
| HW2 | log odds survival = *a* + *b_1_*(immersion time) | 2929.93 | 159.51 | 175.73 |
| HW3 | log odds survival = *a* + *b_1_*(temperature^2^) | 248.94 | 59.30 | 82.34 |
| HW4 | log odds survival = *a* + *b_1_*(temperature) + *b_2_*(immersion time) + *b_3_*(temperature:immersion time) | **137.62** | 65.59 | 87.00 |
| HW5 | log odds survival = *a* + *b_1_*(temperature) + *b_2_*(immersion time) | 168.98 | 64.53 | 87.38 |
| HW6 | log odds survival = *a* + *b_1_*(temperature^2^) + *b_2_*(immersion time) + *b_3_*(temperature^2^: immersion time) | 137.24 | 60.33 | 77.32 |
| HW7 | log odds survival = *a* + *b_1_*(temperature^2^) + *b_2_*(immersion time) | 169.06 | 59.58 | 78.91 |
| HW8 | log odds survival = *a* + *b_1_*(temperature^2^) + *b_2_*(temperature) + *b_3_*(immersion time) | 170.97 | **55.83** | **67.78** |
| **Air-drying experiments** | | **qAIC** | | |
| AD1 | log odds survival = *a* + *b_1_*(drying time) | 53.43 | 113.36 | 152.99 |
| AD2 | log odds survival = *a* + *b_1_*(drying time^2^) + *b_2_*(drying time) | **51.20** | **43.45** | 136.03 |
| AD3 | log odds survival = *a* + *b_1_*(drying time^2^) | 59.10 | 74.57 | **135.72** |
| **Experiments combining hot water and air-drying** | | **qAIC** | | |
| C1 | log odds survival = *a* + *b_1_*(drying time) + *b_2_*(temperature) + *b_3_*(drying time:temperature) | 136.26 | 118.88 | 57.47 |
| C2 | log odds survival = *a* + *b_1_*(drying time) + *b_2_*(temperature) | 136.21 | 119.79 | 58.43 |
| C3 | log odds survival = *a* + *b_1_*(drying time^2^) + *b_2_*(temperature) + *b_3_*(drying time^2^:temperature) | 153.66 | 115.89 | 53.41 |
| C4 | log odds survival = *a* + *b_1_*(drying time) + *b_2_*(temperature^2^) + *b_3_*(drying time:temperature^2^) | 126.78 | 108.38 | 50.92 |
| C5 | log odds survival = *a* + *b_1_*(drying time^2^) + *b_2_*(drying time) + *b_3_*(temperature) | 110.93 | 117.22 | 52.38 |
| C6 | log odds survival = *a* + *b_1_*(temperature^2^) + *b_2_*(temperature) + *b_3_*(drying time) | **108.60** | **77.53** | **30.38** |
| C7 | log odds survival = *a* + *b_1_*(drying time^2^) + *b_2_*(temperature) | 152.22 | 115.28 | 51.60 |
| C8 | log odds survival = *a* + *b_1_*(drying time) + *b_2_*(temperature^2^) | 125.71 | 108.67 | 51.05 |
| C9 | log odds survival = *a* + *b_1_*(drying time) | 190.27 | 261.33 | 154.57 |
| C10 | log odds survival = *a* + *b_1_*(temperature) | 171.43 | 302.36 | 170.08 |
| C11 | log odds survival = *a* + *b_1_*(drying time^2^) | 202.56 | 254.43 | 149.11 |
| C12 | log odds survival = *a* + *b_1_*(temperature^2^) | 162.49 | 291.98 | 163.45 |
| C13 | log odds survival = *a* + *b_1_*(drying time^2^) + *b_2_*(temperature^2^) | 142.53 | 104.40 | 45.00 |
| Note: Models are shown with the link function applied to the response variable; *a* and *b_i_* represent regression coefficients; “:” represents the interaction between variables. Selected models based on qAIC and parsimony are indicated in boldface. | | | | |

| Table S13. Comparison of logistic regression models for acclimation experiments on invertebrates, using qAIC. | | | |
| --- | --- | --- | --- |
| ***Invertebrates*** | | Banded mystery snails | Zebra mussels |
| *Logistic regression models* | | **qAIC** | |
| A1 | log odds survival = *a* + *b_1_*(hot water) + *b_2_*(acclimation) + *b_3_*(hot water:acclimation) | 77.83 | 74.12 |
| A2 | log odds survival = *a* + *b_1_*(hot water) + *b_2_*(acclimation) | 81.15 | 86.06 |
| A3 | log odds survival = *a* + *b_1_*(hot water^2^) + *b_2_*(acclimation) + *b_3_*(hot water^2^:acclimation) | 71.89 | 66.91 |
| A4 | log odds survival = *a* + *b_1_*(hot water^2^) + *b_2_*(hot water) + *b_3_*(acclimation) | **69.65** | **50.71** |
| A5 | log odds survival = *a* + *b_1_*(hot water^2^) + *b_2_*(acclimation) | 74.93 | 73.86 |
| A6 | log odds survival = *a* + *b_1_*(hot water) | 79.65 | 87.99 |
| A7 | log odds survival = *a* + *b_1_*(acclimation) | 151.74 | 519.47 |
| A8 | log odds survival = *a* + *b_1_*(hot water^2^) | 73.47 | 76.30 |
| Note: Models are shown with the link function applied to the response variable; *a* and *b_i_* represent regression coefficients; “:” represents the interaction between variables. Selected models based on qAIC and parsimony are indicated in boldface. | | | |

| Table S14. Comparison of Poisson regression models for experiments on Eurasian watermilfoil and Carolina fanwort. | | | | | |
| --- | --- | --- | --- | --- | --- |
|  | | | Number of leaves | Number of roots | Number of branches |
| **Hot water experiments** | | **Species** | **qAIC** | | |
| HW1 | log count = *a* + *b_1_*(temperature) | EWM | 64.41 | 121.45 | 125.96 |
|  |  | CF | 92.01 | 114.47 | 198.17 |
| HW2 | log count = *a* + *b_1_*(immersion time) | EWM | 168.14 | 224.09 | 224.68 |
|  |  | CF | 176.60 | 233.56 | 180.72 |
| HW3 | log count = *a* + *b_1_*(temperature^2^) | EWM | 52.61 | 114.25 | 116.19 |
|  |  | CF | 86.53 | 108.73 | 185.74 |
| HW4 | log count = *a* + *b_1_*(temperature) + *b_2_*(immersion time) + *b_3_*(temperature:immersion time) | EWM | 66.92 | 122.31 | 127.91 |
|  |  | CF | 94.18 | 116.12 | 200.62 |
| HW5 | log count = *a* + *b_1_*(temperature) + *b_2_*(immersion time) | EWM | 65.36 | 120.38 | 126.13 |
|  |  | CF | 92.43 | 114.17 | 198.64 |
| HW6 | log count = *a* + *b_1_*(temperature^2^) + *b_2_*(immersion time) + *b_3_*(temperature^2^: immersion time) | EWM | 55.05 | 115.14 | 118.48 |
|  |  | CF | 88.77 | 110.37 | 188.19 |
| HW7 | log count = *a* + *b_1_*(temperature)^2^ + *b_2_*(immersion time) | EWM | 53.55 | 113.18 | 116.67 |
|  |  | CF | 86.94 | 108.44 | 186.21 |
| HW8 | log count = *a* + *b_1_*(temperature^2^) + *b_2_*(temperature) + *b_3_*(immersion time) | EWM | 27.75 | 111.06 | 110.13 |
|  |  | CF | 86.07 | 108.83 | 171.81 |
| HW9 | log count = *a* + *b_1_*(temperature^2^) + *b_2_*(immersion time^2^) | EWM | 53.92 | 113.29 | 117.05 |
|  |  | CF | 86.63 | **107.55** | 186.43 |
| HW10 | log count = *a* + *b_1_*(temperature^2^) + *b_2_*(temperature) | EWM | **26.80** | **112.13** | **109.95** |
|  |  | CF | **85.66** | 109.12 | **171.34** |
| **Air-drying experiments** | | **Species** | **qAIC or AIC (*)** | | |
| AD1 | log count = *a* + *b_1_*(drying time) | EWM | **23.40** | **41.66** | **42.79** |
|  |  | CF | **24.74** | 43.12 | 96.48* |
| AD2 | log count = *a* + *b_1_*(drying time^2^) + *b_2_*(drying time) | EWM | 24.68 | 41.06 | 43.88 |
|  |  | CF | 26.69 | 38.00 | 95.87* |
| AD3 | log count = *a* + *b_1_*(drying time^2^) | EWM | 28.34 | 47.82 | 47.01 |
|  |  | CF | 25.23 | **37.97** | **94.10*** |
| **Experiments combining hot water and air-drying** | | **Species** | **qAIC or AIC (*)** | | |
| C1 | log count = *a* + *b_1_*(drying time) + *b_2_*(temperature) + *b_3_*(drying time:temperature) | EWM | 47.35 | 274.10* | 248.95* |
|  |  | CF | **151.75** | 259.23* | 329.90 |
| C2 | log count = *a* + *b_1_*(drying time) + *b_2_*(temperature) | EWM | 47.38 | 284.32* | 255.81* |
|  |  | CF | 161.60 | **281.16*** | 346.94 |
| C3 | log count = *a* + *b_1_*(drying time^2^) + *b_2_*(temperature) + *b_3_*(drying time^2^:temperature) | EWM | 49.19 | 304.92* | 274.00* |
|  |  | CF | 166.61 | 288.14* | 356.02 |
| C4 | log count = *a* + *b_1_*(drying time) + *b_2_*(temperature^2^) + *b_3_*(drying time:temperature^2^) | EWM | 46.82 | 267.31* | 242.89* |
|  |  | CF | 152.16 | 256.78* | **325.61** |
| C5 | log count = *a* + *b_1_*(drying time^2^) + *b_2_*(drying time) + *b_3_*(temperature) | EWM | 49.01 | **267.08*** | **239.81*** |
|  |  | CF | 157.77 | 270.35* | 337.17 |
| C6 | log count = *a* + *b_1_*(temperature^2^) + *b_2_*(temperature) + *b_3_*(drying time) | EWM | 49.13 | 277.50* | 147.73* |
|  |  | CF | 163.59 | 282.54* | 346.30 |
| C7 | log count = *a* + *b_1_*(drying time^2^) + *b_2_*(temperature) | EWM | 50.20 | 312.40* | 278.17* |
|  |  | CF | 175.68 | 307.45* | 368.98 |
| C8 | log count = *a* + *b_1_*(drying time) + *b_2_*(temperature^2^) | EWM | **47.13** | 279.73* | 251.72* |
|  |  | CF | 162.94 | 280.86* | 344.51 |
| C9 | log count = *a* + *b_1_*(drying time) | EWM | 59.17 | 319.79* | 276.88* |
|  |  | CF | 215.56 | 370.17* | 408.34 |
| C10 | log count = *a* + *b_1_*(temperature) | EWM | 59.06 | 356.52* | 309.74* |
|  |  | CF | 205.99 | 359.92* | 413.36 |
| C11 | log count = *a* + *b_1_*(drying time^2^) | EWM | 61.99 | 347.88* | 299.24* |
|  |  | CF | 229.64 | 396.45* | 430.38 |
| C12 | log count = *a* + *b_1_*(temperature^2^) | EWM | 58.80 | 351.93* | 305.64* |
|  |  | CF | 207.33 | 359.62* | 410.93 |
| C13 | log count = *a* + *b_1_*(drying time^2^) + *b_2_*(temperature^2^) | EWM | 49.95 | 307.81* | 274.08* |
|  |  | CF | 177.02 | 307.14* | 366.55 |
| C14 | log count = *a* + *b_1_*(drying time^2^) + *b_2_*(drying time) | EWM | 60.80 | 302.55* | 260.78* |
|  |  | CF | 211.74 | 359.35* | 398.57 |
| C15 | log count = *a* + *b_1_*(temperature^2^) + *b_2_*(temperature) | EWM | 60.80 | 349.70* | 301.65* |
|  |  | CF | 207.98 | 361.30* | 412.72 |
| C16 | log count = *a* + *b_1_*(drying time^2^) + *b_2_*(temperature^2^) + *b_3_*(drying time^2^:temperature^2^) | EWM | 48.85 | 349.70* | 268.63* |
|  |  | CF | 167.64 | 286.96* | 352.40 |
| Note: Models are shown with the link function applied to the response variable; *a* and *b_i_* represent regression coefficients; “:” represents the interaction between variables. Selected models based on AIC/qAIC and parsimony are indicated in boldface. | | | | | |

| Table S15. Comparison of Poisson regression models for acclimation experiments on Eurasian watermilfoil. | | | | |
| --- | --- | --- | --- | --- |
| *Poisson regression models* | | Number of leaves | Number of roots | Number of branches |
|  |  | **qAIC** | | |
| A9 | log odds survival = *a* + *b_1_*(acclimation) + *b_2_*(hot water) + *b_3_*(acclimation:hot water) | 107.65 | 184.09 | 218.66 |
| A10 | log odds survival = *a* + *b_1_*(acclimation) + *b_2_*(hot water) | 105.65 | 182.58 | 216.99 |
| A11 | log count = *a* + *b_1_*(acclimation^2^) + *b_2_*(hot water) + *b_3_*(acclimation^2^:hot water) | 108.48 | 184.59 | 218.90 |
| A12 | log count = *a* + *b_1_*(acclimation) + *b_2_*(hot water^2^) + *b_3_*(acclimation:hot water^2^) | 93.44 | 175.27 | 202.22 |
| A13 | log count = *a* + *b_1_*(acclimation^2^) + *b_2_*(acclimation) + *b_3_*(hot water) | 103.20 | 182.27 | 218.26 |
| A14 | log count = *a* + *b_1_*(hot water^2^) + *b_2_*(hot water) + *b_3_*(acclimation) | **60.93** | 165.12 | 172.17 |
| A15 | log count = *a* + *b_1_*(acclimation^2^) + *b_2_*(hot water) | 106.48 | 182.94 | 217.20 |
| A16 | log count = *a* + *b_1_*(acclimation) + *b_2_*(hot water^2^) | 91.44 | 173.64 | 200.46 |
| A17 | log count = *a* + *b_1_*(acclimation) | 172.11 | 246.29 | 300.90 |
| A18 | log count = *a* + *b_1_*(hot water) | 107.65 | 182.09 | 216.36 |
| A19 | log count = *a* + *b_1_*(acclimation^2^) | 172.94 | 246.65 | 301.10 |
| A20 | log count = *a* + *b_1_*(hot water^2^) | 93.44 | 173.15 | 199.83 |
| A21 | log count = *a* + *b_1_*(acclimation^2^) + *b_2_*(hot water^2^) | 92.27 | 174.00 | 200.67 |
| A22 | log count = *a* + *b_1_*(acclimation^2^) + *b_2_*(acclimation) | 169.66 | 245.98 | 302.12 |
| A23 | log count = *a* + *b_1_*(hot water^2^) + *b_2_*(hot water) | 62.93 | **164.63** | **171.54** |
| A24 | log count = *a* + *b_1_*(acclimation^2^) + *b_2_*(hot water^2^) + *b_3_*(acclimation^2^:hot water^2^) | 94.27 | 175.74 | 202.46 |
| Note: Models are shown with the link function applied to the response variable; *a* and *b_i_* represent regression coefficients; “:” represents the interaction between variables. Selected models based on qAIC and parsimony are indicated in boldface. | | | | |

| Table S16. Comparison of poisson regression models for experiments on European frogbit. | | | |
| --- | --- | --- | --- |
|  | | Number of leaves | Number of turions |
| **Hot water experiments** | | **qAIC** | |
| HW1 | log count = *a* + *b_1_*(temperature) | 284.23 | 613.47 |
| HW2 | log count = *a* + *b_1_*(immersion time) | 303.81 | 670.66 |
| HW3 | log count = *a* + *b_1_*(temperature^2^) | **283.77** | 614.58 |
| HW4 | log count = *a* + *b_1_*(temperature) + *b_2_*(immersion time) + *b_3_*(temperature:immersion time) | 286.76 | 612.35 |
| HW5 | log count = *a* + *b_1_*(temperature) + *b_2_*(immersion time) | 285.35 | **613.35*** |
| HW6 | log count = *a* + *b_1_*(temperature^2^) + *b_2_*(immersion time) + *b_3_*(temperature^2^:immersion time) | 286.31 | 613.58 |
| HW7 | log count = *a* + *b_1_*(temperature^2^) + *b_2_*(immersion time) | 284.89 | 614.45 |
| HW8 | log count = *a* + *b_1_*(temperature^2^) + *b_2_*(temperature) + *b_3_*(immersion time) | 286.89 | 615.34 |
| HW9 | log count = *a* + *b_1_*(temperature^2^) + *b_2_*(immersion time^2^) | 285.45 | 613.61 |
| HW10 | log count = *a* + *b_1_*(temperature^2^) + *b_2_*(temperature) | 285.77 | 615.47 |
| **Air-drying experiments** | | **qAIC** | |
| AD1 | log count = *a* + *b_1_*(drying time) | 194.37 | 364.97 |
| AD2 | log count = *a* + *b_1_*(drying time^2^) + *b_2_*(drying time) | **191.74** | **359.87** |
| AD3 | log count = *a* + *b_1_*(drying time^2^) | 197.58 | 372.67 |
| **Experiments combining hot water and air-drying** | | **qAIC** | |
| C1 | log count = *a* + *b_1_*(drying time) + *b_2_*(temperature) + *b_3_*(drying time:temperature) | 64.14 | 114.24 |
| C2 | log count = *a* + *b_1_*(drying time) + *b_2_*(temperature) | 62.16 | 113.02 |
| C3 | log count = *a* + *b_1_*(drying time^2^) + *b_2_*(temperature) + *b_3_*(drying time^2^:temperature) | 63.62 | 113.14 |
| C4 | log count = *a* + *b_1_*(drying time) + *b_2_*(temperature^2^) + *b_3_*(drying time:temperature^2^) | 63.89 | 114.62 |
| C5 | log count = *a* + *b_1_*(drying time^2^) + *b_2_*(drying time) + *b_3_*(temperature) | 63.65 | 114.94 |
| C6 | log count = *a* + *b_1_*(temperature^2^) + *b_2_*(temperature) + *b_3_*(drying time) | 63.78 | 114.81 |
| C7 | log count = *a* + *b_1_*(drying time^2^) + *b_2_*(temperature) | 61.71 | 113.12 |
| C8 | log count = *a* + *b_1_*(drying time) + *b_2_*(temperature^2^) | 61.91 | 113.38 |
| C9 | log count = *a* + *b_1_*(drying time) | 63.59 | 116.13 |
| C10 | log count = *a* + *b_1_*(temperature) | 62.22 | **111.32** |
| C11 | log count = *a* + *b_1_*(drying time^2^) | 63.14 | 116.23 |
| C12 | log count = *a* + *b_1_*(temperature^2^) | 61.97 | 111.69 |
| C13 | log count = *a* + *b_1_*(drying time^2^) + *b_2_*(temperature^2^) | **61.46** | 113.48 |
| C14 | log count = *a* + *b_1_*(drying time^2^) + *b_2_*(drying time) | 65.08 | 118.05 |
| C15 | log count = *a* + *b_1_*(temperature^2^) + *b_2_*(temperature) | 63.83 | 113.11 |
| C16 | log count = *a* + *b_1_*(drying time^2^) + *b_2_*(temperature^2^) + *b_3_*(drying time^2^:temperature^2^) | 63.35 | 113.46 |
| Note: Models are shown with the link function applied to the response variable; *a* and *b_i_* represent regression coefficients; “:” represents the interaction between variables. Selected models based on qAIC and parsimony are indicated in boldface. Otherwise, model not having the lowest qAIC (*) was selected to avoid overfitting to the data. | | | |

**Selected models for experiments on macrophytes**

| Table S17. Summary of statistical methods and best models for hot water, air-drying, and combined treatment experiments including three macrophyte species. | | |
| --- | --- | --- |
| ***Hot water experiments*** | | |
| Eurasian watermilfoil | *Transformed data, GLM, quasi-Poisson family* | |
|  | Leaf | log count = *a* + *b_1_*(temperature^2^) + *b_2_*(temperature) |
|  | Root |  |
|  | Branch |  |
| Carolina fanwort | *Transformed data, GLM, quasi-Poisson family* | |
|  | Leaf | log count = *a* + *b_1_*(temperature^2^) + *b_2_*(temperature) |
|  | Root | log count = *a* + *b_1_*(temperature^2^) + *b_2_*(time^2^) |
|  | Branch | log count = *a* + *b_1_*(temperature^2^) + *b_2_*(temperature) |
| European frogbit | *Transformed data, GLM, quasi-Poisson family* | |
|  | Leaf | log count = *a* + *b_1_*(temperature^2^) |
|  | Turion | log count = *a* + *b_1_*(temperature) + *b_2_*(time) |
| ***Air-drying experiments*** | | |
| Eurasian watermilfoil | *Transformed data, GLM, quasi-Poisson family* | |
|  | Leaf | log count = *a* + *b_1_*(time) |
|  | Root |  |
|  | Branch |  |
| Carolina fanwort | *Transformed data, GLM, quasi-Poisson family* | |
|  | Leaf | log count = *a* + *b_1_*(time) |
|  | Root | log count = *a* + *b_1_*(time^2^) |
|  | *Transformed data, GLM, Poisson family* | |
|  | Branch | log count = *a* + *b_1_*(time^2^) |
| European frogbit | *Transformed data, GLM, quasi-Poisson family* | |
|  | Leaf | log count = *a* + *b_1_*(time^2^) + *b_2_*(time) |
|  | Turion |  |
| ***Experiments combining hot water and air-drying*** | | |
| Eurasian watermilfoil | *Transformed data, GLM, quasi-Poisson family* | |
|  | Leaf | log count = *a* + *b_1_*(time) + *b_2_*(temperature^2^) |
|  | *Transformed data, GLM, Poisson family* | |
|  | Root | log count = *a* + *b_1_*(time)^2^ + *b_2_*(time) + *b_3_*(temperature) |
|  | Branch |  |
| Carolina fanwort | *Transformed data, GLM, quasi-Poisson family* | |
|  | Leaf | log count = *a* + *b_1_*(time) + *b_2_*(temperature) + *b_3_*(time:temperature) |
|  | Branch | log count = *a* + *b_1_*(time) + *b_2_*(temperature^2^) + *b_3_*(time:temperature^2^) |
|  | *Transformed data, GLM, Poisson family* | |
|  | Root | log count = *a* + *b_1_*(time) + *b_2_*(temperature) |
| European frogbit | Data not transformed, GLM, quasi-Poisson family | |
|  | Leaf | log count = *a* + *b_1_*(time^2^) + *b_2_*(temperature^2^) |
|  | Turion | log count = *a* + *b_1_*(temperature) |
| **Note:** (i) *a* and *b_i_* represent regression coefficients; (ii) “:” represents the interaction between variables; (iii) data was transformed by adding 1 to all counts. | | |
